# Infrastructure and work process in primary health care: PMAQ in Ceará

**DOI:** 10.11606/s1518-8787.2020054001878

**Published:** 2020-06-03

**Authors:** Anya Pimentel Gomes Fernandes Vieira-Meyer, Ana Patrícia Pereira Morais, José Maria Ximenes Guimarães, Isabella Lima Barbosa Campelo, Neiva Francenely Cunha Vieira, Maria de Fátima Antero Sousa Machado, Paula Sacha Frota Nogueira, Sharmênia de Araújo Soares Nuto, Roberto Wagner Júnior Freire de Freitas

**Affiliations:** I Fundação Oswaldo Cruz Ceará EusébioCE Brasil Fundação Oswaldo Cruz Ceará. Eusébio, CE, Brasil; II Universidade Estadual do Ceará Centro de Ciências da Saúde FortalezaCE Brasil Universidade Estadual do Ceará. Centro de Ciências da Saúde. Fortaleza, CE, Brasil; III Centro Universitário Fanor FortalezaCE Brasil Centro Universitário Fanor. Curso de Enfermagem. Fortaleza, CE, Brasil; IV Universidade Federal do Ceará Faculdade de Farmácia, Odontologia e Enfermagem Departamento de Enfermagem FortalezaCE Brasil Universidade Federal do Ceará. Faculdade de Farmácia, Odontologia e Enfermagem. Departamento de Enfermagem. Fortaleza, CE, Brasil; V Universidade Regional do Cariri Centro de Ciências da Saúde Departamento de Enfermagem CratoCE Brasil Universidade Regional do Cariri. Centro de Ciências da Saúde. Departamento de Enfermagem. Crato, CE, Brasil

**Keywords:** Health Care Quality, Access, and Evaluation, Family Health Strategy, Health Infrastructure, Primary Health Care, Outcome and Process Assessment, Health Care

## Abstract

**OBJECTIVE:**

To analyze the quality of the infrastructure and work process of the Family Health Strategy in the municipalities of Ceará between 2012 and 2014.

**METHODS:**

Cross-sectional study, using secondary data from the external evaluation of the 1st (2012) and 2nd (2014) cycle of the National Program for Improvement of Access and Quality of Primary Care in Ceará. A total of 20 composite indicators were used to verify the quality of infrastructure and work process.

**RESULTS:**

Data from 183 (99.4%) of the 184 municipalities of Ceará were collected in both cycles. A total of 1,441 teams were evaluated for the infrastructure and 800 for the work process. Among the 20 composite indicators evaluated, 18 presented an improvement, but in a non-homogeneous way, ranging between 0.0 and 413.5%. We observed that the lower the initial value of the indicator, the greater the variation in quality between 2012 and 2014. The indicators of infrastructure and work process were influenced by the regional health system and population size of the municipality, being more evident the influence on the variables of the work process.

**CONCLUSIONS:**

We identified that quality improvements related to infrastructure and work process occurred in the period of implementation of the program in the state of Ceará in an equitable manner, being influenced by population size and regional health system, showing the influence of the context in the implementation of public policies of this nature.

## INTRODUCTION

In Brazil, in 1988, the Unified Health System (SUS) was created^1^, guided by principles such as universality, equity and integrality, which implies the provision of care within the scope of care networks. Therefore, SUS adopts Primary Health Care (PHC) as central in the structuring of the health system, acting as the first contact of the user and the orderly of care network, according to recommendations of the Declaration of Alma-Ata^2^.

In this context, PHC was instituted based on the concept of comprehensive health care, but with gradual implementation, initially in the form of focused programs aimed to at-risk populations, such as the *Programa de Agentes Comunitários de Saúde* (PACS – Program of Community Health Agents), created in 1991, and the Family Health Program (FHP), established in 1994, which had greater coverage expansion in municipalities with a low human development index (HDI)^3,4^. Therefore, contradictions are shown in the organization of PHC in the early 1990s, with discussions on its traits of selective primary care, highlighting the challenges to advance towards the structuring of comprehensive primary care, necessary for the construction of an integrated health system^2^.

With the normative advance that regulates the organization of SUS, it is evident that FHP was established as a model of health care in 1996, with a redefinition of the funding logic by the implementation of the primary care base (PCB). Later, the program was defined as the Family Health Strategy (FHS) in 2006, with the attribution of acting as a reorganizer of PHC, to promote the integration of different levels of health care, materializing, at the local level, principles and guidelines of the SUS^5^.

The expansion of the FHS throughout the country, over the last 20 years, has been favoring the universalization of primary care and adding basic principles of a comprehensive PHC^6^. In 2014, according to data from the Brazilian Ministry of Health, 5,463 (98%) Brazilian municipalities had Family Health Strategy teams (FHST) in their network, covering 60% of the population. In the same period, 184 municipalities of Ceará had 2,303 FHST implemented, with 77.7% of population coverage^7^.

This increase in coverage occurred heterogeneously in the different regions of Brazil^8^. Thus, challenges are identified to the consolidation of FHS related to the financing, planning and organization of care practices, work management and continuing education of professionals, the coordination of care by the difficulty of ensuring access to other levels of care and construction of the integrality of care, which may compromise the quality of services offered^6^.

Then federal financial and investments aimed to qualify the primary care network are identified, by the guarantee of access and quality of care offered^8^, such as the *Progama Nacional de Melhoria do Acesso e da Qualidade da Atenção Básica* (PMAQ-AB—National Program for Improvement of Access and Quality of Primary Care), established in 2011 by the Brazilian Ministry of Health, being operationalized based on the following phases: adherence, contractualization, development (stage where external evaluation occurs) and recontractualization^10^. PMAQ-AB also represents an institutionalization strategy for quality assessment in PHC, whose evaluation model is based on the triad structure, process and results, proposed by Donabedian^11^.

The option to work with the evaluation of health care in PHC places the researcher in a challenging position, as it requires the choice of policies, actions and territories with various references to Brazil that show efforts and events in the organization of the offer and quality of health services. PMAQ-AB has provided an opportunity for reflections and daily practices of evaluation and self-assessment, which induce improvement planning by teams, thus committing themselves to changes in the infrastructure and work process of the FHS^10^. However, the incorporation of evaluative practices in the daily life of the FHS is still incipient, as well as knowledge about the quality of these services, particularly in the state of Ceará, considered a cutting-edge in PHC actions in the country.

Thus, this study aims to analyze the quality of the FHS infrastructure and work process in the municipalities of Ceará between 2012 and 2014, investigating the existence of quality-mediating variables.

## METHODS

Cross-sectional study using secondary data from the external evaluation of the 1st (2012) and 2nd (2014) cycle of PMAQ-AB referring to the state of Ceará, Brazil. Three instruments focusing on the FHS team are used for this evaluation: module I – referring to infrastructure, with variables observed directly in the health unit; module II – related to the work process, in which the questions are answered by a member of the FHS team (doctor, nurse or dentist); module III – with questions related to user satisfaction, covering their perception and satisfaction regarding access and use of FHS. In this study, only modules I and II (infrastructure and work process) were used. The questionnaires were composed of 450 questions related to infrastructure and 750 related to the work process.

Data collection was coordinated by a group of researchers from universities and research institutions responsible for the external evaluation of PMAQ-AB, who trained and monitored field interviewers and data collection supervisors, including state research coordinators.

In the first evaluation cycle, a total of 184 (100%) municipalities participated, including 911 teams (46.5% of FHST implemented by 2012). In the second cycle, 183 (99.4%) municipalities participated, including 1,711 teams (74.3% of FHST established by 2014). In 2012, more observations on structure than work process were observed, because in 2012 the Brazilian Ministry of Health was particularly interested in infrastructure; therefore, the module referring to this aspect was applied in all basic health units, even in those that did not adhere to the PMAQ-AB. Out of the 184 municipalities, data from 183 (99.4%) whose teams were evaluated in both cycles were used. A total of 1,441 teams were evaluated for the infrastructure and 800 for the work process. Since some FHST did not answer all the questions of modules I and II, the number of teams ranges according to the outcome and year of evaluation.

### Index Creation

The indexes of this study were created based on the national database of the external evaluation of the PMAQ-AB, originally used to evaluate the FHS in Brazil. For this, similar variables included in the 2012 and 2014 cycles were identified. The items were organized into 20 groups (10 for infrastructure and 10 for the work process), based on FHS guidelines^12^ and evaluation themes of the PMAQ-AB^10^, excluding those with more than 5,000 missing observations per year of research. The application of a series of tests validated these structured groupings: pair correlation, Cronbach’s alpha and factor analysis. Additionally, each item was scaled from 0 to 1 (1 = most positive result) and the items within a group were estimated to form the composite index (CI). Moreover, the average of all CI in the general category created three general variables of composite index, two for infrastructure and one for the work process. Table 1 describes the CI created.

**Table 1:** List of variables that composed each composite index created based on the questions of the external evaluation of the National Program for Improvement of Access and Quality of Primary Care in 2012 and 2014.

Compound index name	No. of questions	Description of the questions
Medication	47	Full list of 47 medicines.
Diagnostic tests	4	Test for *Plasmodium* (thick blood smear test); rapid HIV testing; rapid pregnancy test; rapid test for syphilis.
Vaccine	12	Oral rotavirus vaccine; tetravalent (2012) and pentavalent (2014); DTP (diphtheria, tetanus and pertussis); triple viral; 10-valent pneumococcal; pneumococcal (Salk and Sabin); 23-valent pneumococcal; meningococcal C; hepatitis B; seasonal influenza; double adult type dT; BCG ID.
Health attention equip	17	Vaccination card; pregnant woman’s booklet; children’s booklet; tongue lowerer in sufficient quantity; disposable needles of various sizes; bandages; thermal boxes for vaccines; measuring tape; disposable speculum; macrodrops and microdrops serum equipment; endocervical brush; Ayres spatula; adhesive tape, micropore tape and others; blade fixer; gauze; glass blade with frosted side; blade holder or plastic bottle with blade cap; capillary glucose measurement reagent strips; disposable syringes of various sizes; disposable syringes with coupled needle; hard container for disposal of sharps.
Medical equip	21	Adult blood pressure apparatus; child blood pressure apparatus; nebulization apparatus; anthropometric scale of 200 kg; children’s scale; anthropometric ruler; adult’s stethoscope; children’s stethoscope; light focus for gynecological exam; refrigerator for vaccines only; pharmacy-only refrigerator; glucometer; table for gynecological examination with leggings; table for clinical examination; ophthalmoscope; sonar; clinical thermometer; otoscope; monofilament kit for sensitivity test (esthesiometer); clinical lantern; extender cable thermometer.
Informatic equip	10	At least one computer in use; at least one webcam in conditions of use; a set of computer speakers; a stabilizer under conditions of use; at least one microphone in conditions of use; at least one printer in use; at least one TV in conditions of use; internet access; access of the team to Telehealth; room dedicated to the use of the internet.
Space adequacy	17	Sanitary for users (male and female); bathroom for employees; waiting room; vaccine room; doctor’s office; dentist’s office; inhalation room; procedure room; dressing room; observation room; sterilization room; collective activity room; good ventilation and air conditioning; adequate lighting; floors and walls of washable surfaces; good acoustics in health unit; offices with privacy for users.
Services offered	9	Vehicle (house calls and other external activities); meeting the needs of the team by vehicle; medical consultation; nursing consultation; dental consultation; dispensing medicines in the pharmacy; vaccination; user embracement and others.
Facility access	4	Wheelchair-adapted corridors; all external entrances and wheelchair-adapted doors; wheelchairs available to users; bathrooms for people with disabilities.
Unit identification	14	Proper signaling; hours of operation of the health unit according to the recommendations of the Ministry of Health; listing of activities offered by the team available to users; scale of professionals with name and working hours available to users; disclosure to users about BHU’s participation in “*Saúde Mais Perto de Você – Acesso e Qualidade* (PMAQ – Health Closer to You – Access and Quality)”; dissemination of the telephone number of the ombudsman of the Ministry of Health; use of the identification badge by professionals; non-disclosure of the team’s actions to users; opening shifts of the unit (morning, afternoon and evening); opening of the unit on all days of the week (Monday to Friday); offering services on weekends; working during lunch hours.
Infrastructure quality	10	Identification at UBS; accessibility; services offered; adequate space; computer equipment; medical supplies; inputs/materials; vaccines; diagnostic tests; medicines.
Infrastructure quality2	9	Identification at UBS; accessibility; services offered; adequate space; computer equipment; inputs/materials; vaccines; diagnostic tests; medicines.
Link to service	6	Contract with direct public administration; stability at work/obligation to hire; how the person got the job; career plan and salaries; receiving financial incentive or performance bonus; participation of the team in permanent education processes organized by the municipality.
Planning	10	Does the team plan activities on a monthly basis? Does the team perform analysis and monitoring of health information and indicators? Has the team carried out self-evaluation in the last six months? Does the team hold meetings often? Is there a definition of the team’s coverage area? Does the health team have territory maps? Are the records used by the team organized by family? Is there a standard model for filling out the cover sheet of medical records? Is there an electronic record implemented by the health team? Does the team consider the user’s vision for the reorganization and qualification of the work process?
City support	5	Does the team receive support or help for planning and organizing the work process? Does the municipality offer to the health team information that assists in analyzing the population’s health situation? Does the team receive aid or support for discussing data and monitoring the health system? Does the team receive permanent institutional support from the municipality to discuss the work process and help with the identified problems? Does the health team receive help from other professionals to assist and/or support the resolution of complex cases?
Patient welcome	7	Does the team embrace the spontaneous demand in the health unit? Does the team have a user removal service when necessary? Is the health team’s agenda organized for health education groups? Does the team renew revenues for users of continued care or programs such as hypertension and diabetes, without the need to schedule medical appointments? Is there a reservation of vacancies in the schedule or schedule of easy access to the professional so that the user can search and show test results? Is there a reservation of vacancies in the schedule or schedule of easy access to the professional so that the user can answer doubts after consultation or show how they situation has evolved? Does the team forward complaints of visual acuity or refractive evaluation demand, without the need for consultation appointment?
Exams	11	Does the team offer actions for pregnant women? Does the team offer actions for children? Does the team offer actions for patients with diabetes mellitus? Does the health unit perform the creatinine test? Does the health unit perform the lipid profile test? Does the health unit perform the electrocardiogram exam? Does the health unit perform the glycosylated hemoglobin test? Does the health unit perform a bacilloscopy test for tuberculosis? Does the health unit perform chest X-ray exam to diagnose tuberculosis? Does the health unit perform bacilloscopy examination for leprosy? Does the health unit perform the mammography exam?
Prenatal	14	Does the health unit perform the fasting blood glucose test in prenatal care network? Does the health unit perform the syphilis test (VDRL) in prenatal care network? Does the health unit perform HIV tests in the prenatal care network? Does the health unit perform the hepatitis B test in the prenatal care network? Does the health unit perform a summary examination and urine culture in the prenatal care network? Does the team supply the prenatal information system monthly? Does the team use the booklet or card to monitor pregnant women? Is there a record about the professional responsible for monitoring the pregnant woman? Is there a record of the pregnant woman’s dental consultation? Is there a record of the vaccination status of the pregnant woman? Is there a record on the collection of cytopathological exam of the pregnant woman? Does the team guide pregnant women about tetanus vaccines? Does the team receive the exams of pregnant women from the territory in a timely manner to perform necessary interventions? Is penicillin G benzathine applied in the health unit?
Child attention	9	Does the team perform childcare for children up to two years of age? Does the team use the child’s health booklet to monitor growth and development? Does the team have a copy of the child’s health booklets or another form with equivalent information in the unit? In the follow-up of the children of the territory, is there an actualized record on vaccination? In the monitoring of the children of the territory, is there a record on growth and development? In the follow-up of the children of the territory, is there a record on nutritional status? In the monitoring of the children of the territory, is there a record on foot testing? In the monitoring of the children of the territory, is there a record of family violence? In the monitoring of the children of the territory, is there a record on accidents?
Health promotion	12	Does the team offer educational and health promotion actions aimed to women (cervical and breast cancer)? Does the team offer educational and health promotion actions aimed at family planning? Does the team offer educational and health promotion actions aimed to pregnant women and postpartum women (breastfeeding)? Does the team offer educational and health promotion actions aimed to family planning? Does the team offer educational and health promotion actions aimed to older adults? Does the team offer educational and health promotion actions aimed to healthy eating? Does the team offer educational and health promotion actions directed to educational strategies related to sexual health and reproductive health? Does the team conduct groups focused on guidance on communicable diseases (such as dengue, tuberculosis, Hansen’s disease, HIV and trachoma), according to the need of the territory? Does the team conduct groups focused on guiding the use, abuse and dependence from using crack, alcohol and other drugs? Does the team conduct groups guiding the use, abuse and dependence of anxiolytics and benzodiazepines? Does the team address issues related to psychological distress or mental health promotion in the territory? Does the team encourage and develop physical practices and/or physical activities in the basic health unit and/or in the territory?
Home visit	9	Does the team have a protocol or criteria for house calls? Are families in the area covered by the primary care team visited with different frequency, according to risk and vulnerability assessments? Do community health agents have the schedule of visits made according to the priorities of the whole team? Does the team have a survey/mapping of enrolled users who need to receive care at home (except bedridden)? Does the team have a record of the number of bedridden and domiciled in the territory? In home care, do the team professionals perform clinical care (older adult user and/or one who needs home care)? In home care, do team professionals perform nursing procedures? Does the team have communication channels that allow users to express their demands, complaints and/or suggestions in primary care? Is there a local health council or other popular participation spaces?
School health	15	Does the team perform activities at the school? Does the team update the vaccination schedule? Does the team perform early detection of systemic arterial hypertension? Does the team perform detection of neglected health problems? Does the team perform anthropometric evaluation? Does the team perform ophthalmologic evaluation? Does the team perform nutritional assessment? Does the team perform oral health assessment? Does the team perform actions on food security and promotes healthy eating (educational activities on food promotion and healthy lifestyles)? Does the team promote body practices and physical activity in schools? Does the team conduct education for sexual health, reproductive health and prevention of sexually transmitted infections and AIDS? Does the team perform actions to prevent the use of alcohol, tobacco and other drugs? Does the team perform training actions for education professionals to work with health education? Does the team discuss with school teachers? Doesn’t the team perform health promotion and prevention actions?
Work process quality	10	Professional bond; planning; institutional support; user embracement; exams; prenatal care; child health; health promotion; health at school; house calls.

PHCU: primary health care unit

### Statistical Analysis

The level of evaluation is the FHS, but the unit of analysis is the municipality. Thus, the CI of the municipal level was created by the mean of all FHST scores in the municipality. Depending on the size of the municipality, the number of FHST ranged from less than 5 to 120 teams. Comparisons were made to verify whether all CI in both moments were statistically significant, using t-test, one way analysis of variance (ANOVA) and T^2^ Hotelling test. ANOVA and generalized estimated equation (GEE) were used to compare CI in different regions and sizes of municipalities.

Thus, the variation for each CI was created based on the municipality, being calculated as the result of 2014 CI value minus the 2012 CI value. These values were compared based on the region and population size of the municipality. The percentage of change between the 1st and 2nd cycle for each of the CI was also estimated.

### Stratification Variables

Statistical analysis was repeated using two stratification variables: size of the municipality (population) and health region. The municipalities were categorized based on the number of inhabitants, considering the guidelines of the Brazilian Institute of Geography and Statistics (IBGE): level 1 (0 to 5,000), level 2 (5,001 to 10,000), level 3 (10,001 to 20,000), level 4 (20,001 to 50,000) level 5 (50,001 to 100,000), level 6 (100,001 to 500,000), and level 7 (above 500,001)^13^. However, for this study, levels 1 and 2 were grouped. This stratification was adopted to test the hypothesis that there are differences in quality improvement by population size, since the challenges and management capacity differ according to the size of the municipality.

The state of Ceará is divided into five health macro-regions: Fortaleza (44 municipalities), Sobral (54 municipalities), Cariri (45 municipalities), Sertão Central (20 municipalities) and Litoral Leste/Jaguaribe (21 municipalities). We considered as a hypothesis that quality improvements may differ in the regions of the state, due to the intrinsic characteristics of each one. In the process of health regionalization, the macro-regions of Fortaleza, Sobral and Cariri were the first created, considered the most developed centers, with specialized care network and tertiarian reference hospitals in their respective headquarters. The Sertão Central and Litoral Leste/Jaguaribe macro-regions were created in 2011 and 2014, respectively, due to the dismemberment of the Fortaleza macro-region^14^.

## RESULTS

Most of CI (18 out of 20) was significantly better in the second cycle than in the first (Table 2). Only two CI did not change significantly over time, one related to infrastructure (vaccine available in the basic health unit – PHCU), and the other related to the work process (exams).

**Table 2:** Comparison of the composite index variable in the years evaluated. Ceará, 2012 and 2014.

Variables	n^a^	Year	Mean^b^	SD	p^c^	% variation
Infrastructure variables						
Unit identification	1,408	2012	0.54	0.16	< 0.001	16.67
	1,441	2014	0.63	0.13		
Facility access	1,440	2012	0.22	0.29	0.001	136.36
	1,441	2014	0.52	0.34		
Services offered	1,441	2012	0.79	0.14	0.000	13.92
	1,441	2014	0.90	0.11		
Space adequacy	1,428	2012	0.61	0.18	0.000	16.39
	1,441	2014	0.71	0.18		
Informatic equip	1,438	2012	0.16	0.20	0.000	56.25
	1,441	2014	0.25			
Medical equip	448	2012	0.63	0.12	0.000	15.87
	1,441	2014	0.73	0.11		
Health attention equip	1,441	2012	0.88	0.11	0.000	6.81
	1,441	2014	0.94	0.08		
Vaccine	1,441	2012	0.82	0.15	0.421	1.21
	1,441	2014	0.83	0.13		
Diagnostic tests	1,441	2012	0.04	0.12	0.000	413.5
	1,441	2014	0.19	0.34		
Medication	1,441	2012	0.43	0.21	0.000	16.27
	1,441	2014	0.50	0.17		
Infrastructure quality	433	2012	0.51	0.89	0.000	21.56
	1,441	2014	0.62	0.10		
Infrastructure quality2	1,392	2012	0.50	0.09	0.000	22.00
	1,441	2014	0.61	0.11		

Work process variables						
Link to service	782	2012	0.50	0.21	0.000	10.00
	789	2014	0.55	0.21		
Planning	784	2012	0.81	0.11	0.000	6.17
	786	2014	0.86	0.75		
City support	698	2012	0.93	0.15	< 0.018	8.13
	798	2014	0.95	0.15		
Patient welcome	792	2012	0.80	0.18	0.000	8.75
	800	2014	0.87	0.15		
Exams	747	2012	0.96	0.74	0.521	0.00
	800	2014	0.96	0.90		
Prenatal	792	2012	0.87	0.84	0.000	3.44
	800	2014	0.90	0.85		
Child attention	799	2012	0.76	0.15	0.000	11.84
	800	2014	0.85	0.14		
Health promotion	779	2012	0.56	0.20	0.000	25.00
	783	2014	0.70	0.22		
School health	686	2012	0.56	0.24	0.000	42.85
	766	2014	0.80	0.18		
Home visit	797	2012	0.69	0.16	0.000	27.53
	800	2014	0.88	0.13		
Work process quality	553	2012	0.76	0.80	0.000	10.52
	734	2014	0.84	0.76		

SD: standard deviation; PHCU: primary health care unit

^a^ Family health teams evaluated in each composite index. Number of team responses: 15,670 for work process variables and 23,022 for infrastructure.

^b^ Mean value for each composite index (CI), with 1.00 being the maximum value.

^c^ T-test by evaluating whether there is a difference between the CI values between the two years (2012 and 2014).

The percentage of improvement was not homogeneous in all CI investigated, ranging between 0.0 and 413.5% (Table 2). A negative relationship was observed between the percentage of change (between the two PMAQ-AB cycles) and the initial value (referring to 2012) of the variable, in which the lower the initial value of CI turned into the greater the variation in quality between 2012 and 2014. This was observed when all variables were analyzed together (r = -0.4843; p = 0.0192). When the infrastructure and work process variables were evaluated separately, only the set of work process variables demonstrated this statistically significant negative relationship (infrastructure: r = -0.4624 and p = 0.1785; work process: r = -0.7031 and p = 0.0233).

When studying the CI values for different regions and population size of the municipalities, we observed that, generally, the variables presented improvements in the mean quality of the municipalities in the period, affecting the quality of PHC results (Table 3 and Table 4).

**Table 3:** Comparison of the values of the variables of infrastructure and work process, according to the population size of the municipalities with external evaluation of the National Program for Improvement of Access and Quality of Primary Care. Ceará, 2012 and 2014.

Infrastructure variables by municipality population size						Work process variables by population size of the municipality					
	
Variable	Population size (in inhabitants)	2012	2014	% change	p^a^	Variable	Population size (in inhabitants)	2012	2014	% change	p^a^
Unit identification	0–10,000	0.542	0.633	16.78		Home visit	0–10,000	0.663	0.848	27.90	
	10,001–20,000	0.524	0.624	19.08			10,001–20,000	0.682	0.875	28.29	
	20,001–50,000	0.532	0.641	20.48			20,001–50,000	0.680	0.868	27.64	
	50,001–100,000	0.528	0.609	15.34	0.688		50,001–100,000	0.697	0.884	26.82	0.235
	100,001–500,000	0.587	0.686	16.86			100,001–500,000	0.741	0.931	25.64	
	≥ 500,001	0.534	0.600	12.35			≥ 500,001	0.707	0.740	4.67	
Facility access	0–10,000	0.270	0.632	134.07		School health	0–10,000	0.633	0.795	25.59	
	10,001–20,000	0.200	0.517	158.50			10,001–20,000	0.605	0.808	33.55	
	20,001–50,000	0.194	0.499	157.21			20,001–50,000	0.558	0.796	42.65	
	50,001–100,000	0.218	0.496	127.52	0.256		50,001–100,000	0.440	0.771	75.23	0.137
	100,001–500,000	0.279	0.559	100.35			100,001–500,000	0.553	0.858	55.15	
	≥ 500,001	0.233	0.600	157.51			≥ 500,001	0.577	0.555	-3.81	
Services offered	0–10,000	0.831	0.926	11.43		Health promotion	0–10,000	0.615	0.638	3.74	
	10,001–20,000	0.770	0.900	16.88			10,001–20,000	0.571	0.684	19.79	
	20,001–50,000	0.797	0.898	12.67			20,001–50,000	0.556	0.708	27.34	
	50,001–100,000	0.769	0.899	16.90	0.562		50,001–100,000	0.504	0.676	34.13	0.971
	100,001–500,000	0.815	0.918	12.63			100,001–500,000	0.641	0.781	21.84	
	≥ 500,001	0.866	0.895	3.34			≥ 500,001	0.569	0.354	-37.78	
Space adequacy	0–10,000	0.616	0.717	16.39		Link to service	0–10,000	0.419	0.462	10.26	
	10,001–20,000	0.579	0.683	17.96			10,001–20,000	0.423	0.493	16.55	
	20,001–50,000	0.584	0.725	24.14			20,001–50,000	0.548	0.600	9.49	
	50,001–100,000	0.625	0.681	8.96	0.285		50,001–100,000	0.498	0.536	7.63	0.001
	100,001–500,000	0.653	0.762	16.69			100,001–500,000	0.473	0.552	16.70	
	≥ 500,001	0.718	0.658	-8.35			≥ 500,001	0.823	0.770	-6.43	
Informatic equip	0–10,000	0.220	0.328	49.09		Planning	0–10,000	0.809	0.857	5.93	
	10,001–20,000	0.103	0.227	120.38			10,001–20,000	0.809	0.852	5.31	
	20,001–50,000	0.126	0.243	92.85			20,001–50,000	0.818	0.868	6.11	
	50,001–100,000	0.140	0.220	57.14	0.920		50,001–100,000	0.775	0.851	9.80	0.688
	100,001–500,000	0.311	0.347	11.57			100,001–500,000	0.855	0.891	4.21	
	≥ 500,001	0.396	0.390	-1.51			≥ 500,001	0.858	0.663	-22.72	
Medical equip	0–10,000	0.650	0.767	18.00		City support	0–10,000	0.930	0.963	3.55	
	10,001–20,000	0.611	0.720	17.83			10,001–20,000	0.923	0.946	2.49	
	20,001–50,000	0.643	0.732	13.84			20,001–50,000	0.948	0.960	1.26	
	50,001–100,000	0.622	0.708	13.82	0.980		50,001–100,000	0.910	0.952	4.61	0.407
	100,001–500,000	0.692	0.780	12.71			100,001–500,000	0.937	0.986	5.23	
	≥ 500,001	0.780	0.717	-8.07			≥ 500,001	0.758	0.418	-44.85	
Health attention equip	0–10,000	0.889	0.945	6.29		Patient welcome	0–10,000	0.818	0.878	7.33	
	10,001–20,000	0.872	0.939	7.68			10,001–20,000	0.809	0.860	6.30	
	20,001–50,000	0.880	0.944	7.27			20,001–50,000	0.805	0.866	7.58	
	50,001–100,000	0.877	0.938	6.95	0.725		50,001–100,000	0.757	0.857	13.21	0.212
	100,001–500,000	0.911	0.954	4.72			100,001–500,000	0.811	0.905	11.59	
	≥ 500,001	0.875	0.932	6.51			≥ 500,001	0.845	0.678	-19.76	
Vaccine	0–10,000	0.829	0.833	0.48		Exams	0–10,000	0.931	0.947	1.72	
	10,001–20,000	0.825	0.831	0.72			10,001–20,000	0.951	0.942	-0.95	
	20,001–50,000	0.796	0.821	3.14			20,001–50,000	0.958	0.957	-0.10	
	50,001–100,000	0.826	0.814	-1.45	0.996		50,001–100,000	0.954	0.955	0.10	0.037
	100,001–500,000	0.853	0.852	-0.11			100,001–500,000	0.986	0.986	0	
	≥ 500,001	0.891	0.801	-10.10			≥ 500,001	0.992	0.893	-9.98	
Diagnostic tests	0–10,000	0.034	0.091	167.64		Prenatal	0–10,000	0.872	0.905	3.78	
	10,001–20,000	0.012	0.158	1216.67			10,001–20,000	0.872	0.904	3.67	
	20,001–50,000	0.030	0.067	123.33			20,001–50,000	0.857	0.901	5.13	
	50,001–100,000	0.015	0.246	1540.00	0.036		50,001–100,000	0.861	0.888	3.13	0.693
	100,001–500,000	0.112	0.460	310.71			100,001–500,000	0.901	0.928	2.99	
	≥ 500,001	0.105	0.305	190.47			≥ 500,001	0.892	0.815	-8.63	
Medication	0–10,000	0.443	0.462	4.28		Child attention	0–10,000	0.730	0.774	6.03	
	10,001–20,000	0.439	0.465	9.92			10,001–20,000	0.744	0.851	14.38	
	20,001–50,000	0.449	0.502	11.80			20,001–50,000	0.751	0.843	12.25	
	50,001–100,000	0.425	0.480	12.94	0.696		50,001–100,000	0.718	0.807	12.39	0.036
	100,001–500,000	0.393	0.546	38.93			100,001–500,000	0.834	0.935	12.11	
	≥ 500,001	0.154	0.598	288.31			≥ 500,001	0.842	0.777	-7.71	
Infrastructure quality	0–10,000	0.530	0.633	19.43		Work process quality	0–10,000	0.754	0.822	9.02	
	10,001–20,000	0.489	0.606	23.92			10,001–20,000	0.752	0.824	9.57	
	20,001–50,000	0.507	0.607	19.72	0.374		20,001–50,000	0.763	0.839	9.96	
	50,001–100,000	0.513	0.609	18.71			50,001–100,000	0.725	0.822	13.38	0.338
	100,001–500,000	0.579	0.686	18.48			100,001–500,000	0.794	0.876	10.33	
	≥ 500,001	0.603	0.650	7.79			≥ 500,001	0.795	0.702	-11.69	
Infrastructure quality2	0–10,000	0.524	0.619	18.12							
	10,001–20,000	0.480	0.594	23.75							
	20,001–50,000	0.487	0.593	21.76	0.846						
	50,001–100,000	0.491	0.598	21.79							
	100,001–500,000	0.541	0.676	24.95							
	≥ 500,001	0.527	0.642	21.82							

PHCU: primary health care unit

^a^ ANOVA – Equations of generalized estimative.

**Table 4:** Comparison of the values of the infrastructure variables and work process, according to the health region in the external evaluation of the National Program for Improvement of Access and Quality of Primary Care. Ceará, 2012 and 2014.

Infrastructure variables by region						Work process variables by region					
	
Variable	Health region	2012	2014	% change	p^a^	Variable	Health region	2012	2014	% change	p^a^
Unit identification	Fortaleza	0.510	0.617	20.98		Link to service	Fortaleza	0.485	0.558	15.05	
	Sobral	0.494	0.596	20.64			Sobral	0.526	0.531	0.95	
	Cariri	0.618	0.685	10.84	0.002		Cariri	0.514	0.590	14.78	0.673
	Sertão Central	0.476	0.609	27.94			Sertão Central	0.371	0.479	29.11	
	East Coast/Jaguaribe	0.583	0.677	16.12			East Coast/Jaguaribe	0.514	0.532	3.50	
Facility access	Fortaleza	0.253	0.525	107.50		Planning	Fortaleza	0.786	0.844	7.37	
	Sobral	0.184	0.475	158.15			Sobral	0.809	0.858	6.05	
	Cariri	0.214	0.571	166.82	0.894		Cariri	0.837	0.878	4.89	0.016
	Sertão Central	0.155	0.501	223.22			Sertão Central	0.787	0.878	11.56	
	East Coast/Jaguaribe	0.261	0.461	76.62			East Coast/Jaguaribe	0.846	0.847	0.11	
Services offered	Fortaleza	0.786	0.903	14.88		City support	Fortaleza	0.889	0.915	2.92	
	Sobral	0.780	0.890	14.10			Sobral	0.952	0.969	1.78	
	Cariri	0.812	0.916	12.80	0.503		Cariri	0.937	0.969	3.41	0.043
	Sertão Central	0.745	0.917	23.08			Sertão Central	0.936	0.953	1.81	
	East Coast/Jaguaribe	0.788	0.880	11.67			East Coast/Jaguaribe	0.948	0.968	2.10	
Space adequacy	Fortaleza	0.639	0.701	9.70		Patient welcome	Fortaleza	0.803	0.844	5.10	
	Sobral	0.594	0.688	15.82			Sobral	0.771	0.837	8.56	
	Cariri	0.582	0.708	21.64			Cariri	0.778	0.906	16.45	
	Sertão Central	0.597	0.649	8.71			Sertão Central	0.809	0.936	15.69	
	East Coast/Jaguaribe	0.191	0.243	27.22			East Coast/Jaguaribe	0.969	0.944	-2.57	
Informatic equip	Fortaleza	0.151	0.263	74.17		Exams	Fortaleza	0.956	0.952	-0.41	
	Sobral	0.134	0.265	97.76	0.266		Sobral	0.967	0.980	1.34	0.310
	Cariri	0.141	0.250	77.30			Cariri	0.930	0.961	3.33	
	Sertão Central	0.186	0.256	37.63			Sertão Central	0.946	0.935	-1.16	
	East Coast/Jaguaribe	0.653	0.720	10.26			East Coast/Jaguaribe	0.879	0.902	2.61	
Medical equip	Fortaleza	0.626	0.710	13.41		Prenatal	Fortaleza	0.879	0.902	2.61	
	Sobral	0.644	0.754	17.08	0.017		Sobral	0.853	0.891	4.45	0.568
	Cariri	0.567	0.719	26.80			Cariri	0.867	0.909	4.84	
	Sertão Central	0.657	0.773	17.65			Sertão Central	0.870	0.924	6.20	
	East Coast/Jaguaribe	0.894	0.936	4.69			East Coast/Jaguaribe	0.776	0.861	10.95	
Health attention equip	Fortaleza	0.883	0.941	6.56		Child attention	Fortaleza	0.756	0.833	10.18	
	Sobral	0.887	0.958	8.00	0.363		Sobral	0.767	0.862	12.38	0.058
	Cariri	0.840	0.930	10.71			Cariri	0.704	0.850	20.73	
	Sertão Central	0.877	0.938	6.95			Sertão Central	0.706	0.792	12.18	
	East Coast/Jaguaribe	0.840	0.838	-0.23			East Coast/Jaguaribe	0.514	0.664	29.18	
Vaccine	Fortaleza	0.788	0.797	1.14		Health promotion	Fortaleza	0.610	0.693	13.60	
	Sobral	0.839	0.830	-1.07	0.666		Sobral	0.608	0.718	18.09	0.023
	Cariri	0.798	0.840	5.26			Cariri	0.463	0.733	58.31	
	Sertão Central	0.818	0.827	1.10			Sertão Central	0.556	0.751	35.07	
	East Coast/Jaguaribe	0.039	0.200	412.82			East Coast/Jaguaribe	0.524	0.772	47.32	
Diagnostic tests	Fortaleza	0.047	0.234	397.87		School health	Fortaleza	0.561	0.766	36.54	
	Sobral	0.025	0.085	240.00	0.497		Sobral	0.615	0.826	34.30	0.001
	Cariri	0.016	0.246	1.437.50			Cariri	0.493	0.829	68.15	
	Sertão Central	0.058	0.352	506.89			Sertão Central	0.579	0.849	46.63	
	East Coast/Jaguaribe	0.449	0.495	10.24			East Coast/Jaguaribe	0.706	0.882	24.92	
Medication	Fortaleza	0.353	0.406	15.01		Home visit	Fortaleza	0.669	0.834	24.66	
	Sobral	0.428	0.517	20.79	0.045		Sobral	0.692	0.896	29.47	0.042
	Cariri	0.447	0.498	11.40			Cariri	0.695	0.910	30.93	
	Sertão Central	0.499	0.498	-0.20			Sertão Central	0.727	0.921	26.68	
	East Coast/Jaguaribe	0.534	0.618	15.73			East Coast/Jaguaribe	0.750	0.822	9.60	
Infrastructure quality	Fortaleza	0.482	0.607	25.93		Work process quality	Fortaleza	0.772	0.823	6.60	
	Sobral	0.523	0.634	21.22	0.160		Sobral	0.771	0.851	10.37	0.171
	Cariri	0.481	0.622	29.31			Cariri	0.711	0.848	19.26	
	Sertão Central	0.531	0.631	18.83			Sertão Central	0.757	0.844	11.49	
	East Coast/Jaguaribe	0.509	0.606	19.05			East Coast/Jaguaribe	0.561	0.766	36.54	
Infrastructure quality2	Fortaleza	0.475	0.595	25.26							
	Sobral	0.505	0.620	22.77	0.154						
	Cariri	0.467	0.611	30.83							
	Sertão Central	0.519	0.615	18.49							
	East Coast/Jaguaribe	0.047	0.234	397.87							

PHCU: primary health care unit

^a^ ANOVA – Equations of generalized estimative

When observing quality changes in the infrastructure, based on the size of the municipality, only the CI of diagnostic tests presented significantly different percentages of change between groups (p = 0.036), with a greater positive effect on quality improvement in municipalities with a population of 10,001 to 20,000 inhabitants and 50,001 to 100,000 inhabitants. These data show that municipality size may not influence the improvement of the quality of infrastructure variables during the period studied.

We found that few infrastructure CI were influenced by the health region of the municipality, with only three presenting statistically significant changes: unit identification (p = 0.002), medical equip (p=0.017) and medication (p = 0.045). However, although no statistically significant differences were found between health regions, we observed that the Sertão Central region showed a higher percentage of improvements in most of the infrastructure variables analyzed, namely: unit identification, facility access, services offered, informatic equip, medical equip, health attention equip, vaccine, diagnostic tests, medication and infrastructure quality and infrastructure equality 2 (Table 4).

When observing the CI variables related to the work process, the different population sizes of the municipality were significantly associated with the change in quality improvement in three CI evaluated, in relation to link to service (p = 0.001), exams (p = 0.037) and child attention (p = 0.036). Notably, although it is not always a statistically significant result, the variables health promotion, school health, planning, patient welcome, and work process quality presented greater percentage variation in municipalities from 50,001 to 100,000 inhabitants. Municipalities with a population above 500,000 inhabitants presented negative variation over the years in all CI of the work process, except for home visit (Table 3).

When observing the changes in the work process by region (Table 4 a significant variation was found among them in six CI studied: planning (p = 0.016), city support (p = 0.043), patient welcome (p = 0.001), health promotion (p = 0.023), school health (p = 0.001) and home visit (p = 0.042). The highest increase in CI occurred in the Sertão Central region, with greater positive variation between regions and in 9 of the 11 CI studied, while Litoral Leste/Jaguaribe and Cariri presented higher variations in CI. It is interesting to note that the CI values in 2012 for the Sertão Central region were, in general, the lowest among the different regions.

## DISCUSSION

This is the first article evaluating, by composite indexes, the quality of FHS in the state of Ceará, in the dimensions of infrastructure and work process, using data from the external evaluation of the PMAQ-AB of the 1st and 2nd cycle (2012 and 2014). In general, a positive variation in the CI of infrastructure and work process was observed (significant change in 18 of the 20 CI evaluated), which indicates improvement in the quality of the FHS in the period studied. We also verified that this improvement occurred more intensely and in an inverse relationship between CI result in 2012 and the percentage of change occurred (difference in values between 2014 and 2012) – that is, the lower the value in 2012, the greater the improvement of the variable. This fact shows a desirable equitable improvement of CI in the period.

This performance reflects, to some extent, the induction performed by evaluation and monitoring policies, with increased investments and adequate use of resources to meet PHC demands^8,15^, as well as the program for requalification of the infrastructure of basic health units of the country (*Requalifica UBS*–Requalifies BHU)^16^. A similar study conducted throughout Brazil also showed a fair improvement in the indicators analyzed, especially when evaluating the North and Northeast regions^17^. Notably, unlike the nationwide study, which presented a more prominent inverse relationship in CI related to infrastructure^17^, in this study such relationship was stronger in CI related to work processes. This may mean that, in general, the *Requalifica UBS*^16^ was effective, but that, in the state of Ceará, the performance of the teams, as well as the management processes linked to them, was able to respond more strongly than in other regions of the country regarding work processes.

It should be noted that this most prominent inverse relationship in CI related to the work process may be the result of the protagonism of the teams, who have worked these questions more effectively, minimizing the differences in quality between the variables studied and qualifying the work of FHS more equitably. Generally, teams have more autonomy to act on problems related to the work process than in the infrastructure dimension, which demands financial resources that are not always available^12,15^. Thus, we observed that the teams participating in the PMAQ-AB effectively expanded their scope of practices, supported by municipal management. Thus, they advanced in the changes related to the work process to qualify the FHS, assuming this transformation process, performing self-assessment and planning, setting goals to be implemented jointly by the teams.

The availability of financial resources has the capacity to induce more rapid improvements in infrastructure, while the transformations of the work process require more time, since they require changes in organizational culture, co-responsibility of managers and professionals, in addition to the reorganization of health practices^18^. The fact that the municipalities of Ceará have succeeded in making progress in the organization of work processes of their FHST may be a reflection of the state performance. Its role is associated with the processes of continuing education and the monitoring of indicators developed, and these actions are executed as a strategy for consolidating the regionalization process^14^. Thus, with the PMAQ, Brazil assumes the responsibility of properly managing the offer of services, so that the results achieved correspond to the established goals or the real needs of the population with a programmed incentive policy^10^.

The lowest percentages of change occurred in the variables exams, vaccine and prenatal, which are among the CI with higher initial values. Furthermore, it is important to understand that the supply of vaccines has its logistics structure organized nationally by the *Programa Nacional de Imunização* (PNI – National Immunization Program )^16^. Thus, immunobiologicals are acquired by the federal government, and the local/municipal level has to adequate the units according to the technical standards of the Ministry of Health and application in the population^19,20^. Thus, the municipalities have low interference in this variable, which can be verified by the non-influence of population size and health region in this CI.

It is interesting to note that the greatest increase in CI occurred in diagnostic tests, a variable in which municipalities also have little influence. The acquisition of the tests is carried out by the Brazilian Ministry of Health, but it is up to the states and municipalities to structure and organize them effectively. The implementation of rapid tests and exams for the diagnosis of pregnancy, HIV infection and screening of syphilis and viral hepatitis in PHC forms the set of strategies of the Ministry of Health aiming to qualify and to expand the Brazilian population’s access to health^21^. The data show that a significant improvement in this indicator occurred in the state, most likely due to the increase in the acquisition of inputs by the federal government, but also by the better organization of the state and municipalities in the distribution and use of such inputs. However, unlike the variable vaccines, the population size and the health region influenced the improvement in diagnostic tests. The explanation for this is not very clear, but it seems to us to be related to the way in which these municipalities organize themselves to carry out the diagnostic tests.

We also observed that the variation of CI did not occur homogeneously in the groups studied, which seems to be influenced by population size and regionalization. The largest variations occurred in the smaller population municipalities, located in the Sertão Central region and with lower CI values in the first external evaluation cycle.

In the process of implementing the Sertão Central macro-region, in 2011, being the penult installed in the state of Ceará^14^, possibly its organization – by the construction of the *Plano Diretor Regional*, which was possible with workshops, meetings, training and agreements between municipal managers and state manager – has mobilized efforts of leaders and professionals aimed to the qualification of their work processes and infrastructure, reflecting in the best CI of the region in the 2nd cycle of the PMAQ. This fact corroborates the effect of regional issues on the implementation of national policies.

We emphasized that mediating variables should be considered in the implementation of public health policies. Inter- and intra-regional differences in health systems can occur for several reasons, whether economic, cultural, educational, organizational, infrastructure-related or population profile, including the epidemiological and demographic^17^.

In municipalities with more than 500,000 inhabitants, such the state capital, a negative variation was observed in CI results regarding the quality of the work process. This fact may be the effect of the organizational change implemented in PHC of the municipality since 2013, notably in the work process of the FHST. In this context, the following stand out: changes in basic health units managers; change in the work day of professionals, who began a work shift of six direct hours, generating mismatches between team members; discouraged local planning in the FHS; changes in the regulation of users’ access, with a dense schedule of care due to spontaneous demand; among other^22,23^. Such modifications seem to have, to some extent, disarticulated the FHST, distancing them from what is recommended by the *Política Nacional de Atenção Básica* (PNAB – National Primary Health Care Policy)^12^. Previous studies have shown a relationship between human development index (HDI), FHS coverage, *Bolsa Família* Program coverage, population size, FHS planning indicators and institutional support for FHS actions and provision of prenatal care and FHS exams as variables that influence health indicators^24-26^. Therefore, for the improvement of the quality of health care, it is necessary to undertake efforts aimed to planning and institutional support, aligned with the organizational mission, considering the interests of the collective of workers, with a view to ensuring the provision of services and resolutive actions.

Considering the fact that the greatest positive variation in quality improvement occurred in municipalities and/or regions with lower initial CI, the implementation of PMAQ-AB in Ceará induced the qualification of the FHS in an equitable manner. In fact, this characteristic also occurs in the rest of the country, and in other policies based on the principle of equity, such as the PNAB, which has also provided a reduction in inequalities, benefiting poorer, smaller and low population-density municipalities^17,24,27^.

The implementation of the PMAQ-AB required greater leadership of managers and workers in the restructuring of basic health units and work processes in the FHS than traditionally occurred in Brazilian states. The standards of access and quality are re-signified according to the concrete reality, context, priorities, interests and negotiation with local actors^8,28^. In this sense, health policies that induce evaluation and monitoring also influence the context in which^29^they are implemented, and they should be considered in the implantation of national public policies, but with local implementation. We believed that part of the differences observed between health regions and population size may have been due to regional issues. Thus, for the full implantation of national policies, additional support is necessary for regions that need greater incentive to achieve quality improvement. Although the analysis of this research occurred in the state of Ceará, its inferences, related to the importance of context issues in the implementation of policies, can be extrapolated to other parts of the country.

The study recognizes that, by the evaluated CI, PMAQ-AB, although recent, promotes the responsibility to adequately manage the provision of services so that the established goals and the real health needs of the population are met and achieved with a programmed incentive policy that directly affects the financing, management of the service network, institutional support, planning and organization of work processes.

## CONCLUSION

Quality improvements related to infrastructure and work process occurred equitably during the implementation period of the PMAQ-AB in the state of Ceará. Although the implementation of the program occurred almost universally among the municipalities of the state, the results of this policy were not homogeneous, since they were influenced both by population size and health region. We observed that public policies are appropriate and adapted according to the reality and/or context in which they are implemented, with the flexibility of considering dynamics and complexity of the territories. Thus, these aspects should be considered when national policies are implemented locally.
