# Supplementary material for: Infrastructure and work process in primary health care: PMAQ in Ceará
Source: Rev Saude Publica. 2020 Jun 3;54:62. doi: 10.11606/s1518-8787.2020054001878 (PMC7274211; doi:10.11606/s1518-8787.2020054001878)
Supplement: Translation

# Translation: Infraestrutura e processo de trabalho na atenção primária à saúde: PMAQ no Ceará

**Authors:** Anya Pimentel Gomes Fernandes Vieira-Meyer, Ana Patrícia Pereira Morais, José Maria Ximenes Guimarães, Isabella Lima Barbosa Campelo, Neiva Francenely Cunha Vieira, Maria de Fátima Antero Sousa Machado, Paula Sacha Frota Nogueira, Sharmênia de Araújo Soares Nuto, Roberto Wagner Júnior Freire de Freitas

**Affiliations:** I Brasil Fundação Oswaldo Cruz Ceará. Eusébio, CE, Brasil; II Brasil Universidade Estadual do Ceará. Centro de Ciências da Saúde. Fortaleza, CE, Brasil; III Brasil Centro Universitário Fanor. Curso de Enfermagem. Fortaleza, CE, Brasil; IV Brasil Universidade Federal do Ceará. Faculdade de Farmácia, Odontologia e Enfermagem. Departamento de Enfermagem. Fortaleza, CE, Brasil; V Brasil Universidade Regional do Cariri. Centro de Ciências da Saúde. Departamento de Enfermagem. Crato, CE, Brasil

**Keywords:** Qualidade, Acesso e Avaliação da Assistência à Saúde, Estratégia Saúde da Família, Infraestrutura Sanitária, Atenção Primária à Saúde, Avaliação de Processos e Resultados (Cuidados de Saúde

## Abstract

**OBJETIVO:**

Analisar a qualidade da infraestrutura e do processo de trabalho da Estratégia Saúde da Família nos municípios do Ceará entre 2012 e 2014.

**MÉTODOS:**

Estudo transversal, utilizando dados secundários da avaliação externa do 1º (2012) e 2º (2014) ciclo do Programa Nacional de Melhoria do Acesso e da Qualidade da Atenção Básica no Ceará. Vinte indicadores compostos foram utilizados para averiguar a qualidade da infraestrutura e do processo de trabalho.

**RESULTADOS:**

Dados de 183 (99,4%) dos 184 municípios foram avaliados nos dois ciclos. Avaliaram-se 1.441 equipes para infraestrutura e 800 equipes para processo de trabalho. Dentre os 20 indicadores compostos avaliados, houve melhoria em 18, porém de forma não homogênea, variando de 0,0 a 413,5%. Observou-se que quanto menor o valor inicial do indicador, maior a variação na qualidade entre 2012 e 2014. Os indicadores da infraestrutura e do processo de trabalho foram influenciados pela região de saúde e porte populacional do município, sendo mais evidente a influência nas variáveis de processo de trabalho.

**CONCLUSÕES:**

Identificou-se que melhorias da qualidade referentes à infraestrutura e ao processo de trabalho ocorreram no período de implantação do programa no estado do Ceará de forma equitativa, sendo influenciadas pelo porte populacional e pela região, demonstrando a influência do contexto na implementação de políticas públicas dessa natureza.

## INTRODUÇÃO

No Brasil, em 1988, foi criado o Sistema Único de Saúde (SUS)^1^, orientado por princípios como universalidade, equidade e integralidade, o que implica a oferta de cuidados nos âmbitos das redes de atenção. Para tanto, ele adota a atenção primária à saúde (APS) como central na estruturação do sistema de saúde, devendo funcionar como primeiro contato do usuário e ordenadora da rede de atenção, de acordo com recomendações da Declaração de Alma-Ata^2^.

Nesse contexto, a APS foi instituída com base na concepção de atenção integral à saúde, porém com implantação gradual, inicialmente sob a forma de programas focalizados voltados às populações de risco, tais como o Programa de Agentes Comunitários de Saúde (PACS), criado em 1991, e o Programa Saúde da Família (PSF), instituído em 1994, que teve maior expansão de cobertura em municípios com baixo índice de desenvolvimento humano (IDH)^3,4^. Evidenciam-se, portanto, contradições na organização da APS no início da década de 1990, com debates sobre seus traços de atenção primária seletiva, ressaltando-se os desafios para avançar rumo à estruturação de uma atenção primária abrangente, necessária à construção de um sistema de saúde integrado^2^.

Com o avanço normativo que regulamenta a organização do SUS, evidencia-se que o PSF foi estabelecido como modelo de atenção à saúde, em 1996, com redefinição da lógica de financiamento por meio da implantação do piso da atenção básica (PAB). Posteriormente, o programa foi definido como Estratégia Saúde da Família (ESF), em 2006, com a atribuição de atuar como reorganizador da APS, com vista a promover a integração dos distintos níveis de atenção à saúde, materializando, no âmbito local, os princípios e diretrizes do SUS^5^.

Observa-se, ao longo dos últimos 20 anos, a expansão da ESF em todo o país, favorecendo a universalização dos cuidados primários e agregando princípios basilares de uma APS abrangente^6^. Em 2014, segundo dados do Ministério da Saúde, 5.463 (98%) municípios brasileiros dispunham de equipes da Estratégia Saúde da Família (EqSF) na sua rede, com cobertura de 60% da população. No mesmo período, os 184 municípios do Ceará contavam com 2.303 EqSF implantadas, com 77,7% de cobertura populacional^7^.

Esse aumento da cobertura ocorreu de modo heterogêneo nas distintas regiões do Brasil^8^. Assim, identificam-se desafios à consolidação da ESF relacionados ao financiamento, ao planejamento e à organização das práticas de cuidado, à gestão do trabalho e à educação permanente dos profissionais, à coordenação do cuidado mediante a dificuldade de garantia do acesso aos demais níveis de atenção e à construção da integralidade da atenção, os quais podem comprometer a qualidade dos serviços ofertados^6^.

Destarte, identificam-se iniciativas e investimentos financeiros federais voltados à qualificação da rede de atenção primária, por meio da garantia do acesso e da qualidade do cuidado ofertado^8,9^, a exemplo do Programa Nacional de Melhoria do Acesso e da Qualidade da Atenção Básica (PMAQ-AB), instituído em 2011 pelo Ministério da Saúde, sendo operacionalizado com base nas seguintes fases: adesão, contratualização, desenvolvimento (etapa em que ocorre a avaliação externa) e recontratualização^10^. O PMAQ-AB representa, ainda, uma estratégia de institucionalização de avaliação da qualidade na APS, cujo modelo avaliativo é pautado na tríade estrutura, processo e resultados, proposta por Donabedian^11^.

A opção de trabalhar com a avaliação de cuidados de saúde na APS coloca o investigador em posição desafiadora, pois exige a escolha de políticas, ações e territórios com várias referências para o Brasil que demonstrem esforços e eventos na organização da oferta e da qualidade dos serviços de saúde. O PMAQ-AB tem oportunizado reflexões e práticas cotidianas de avaliação e autoavaliação, que induzem planejamento de melhorias pelas equipes, comprometendo-se assim com mudanças na infraestrutura e no processo de trabalho da ESF^10^. Entretanto, percebe-se que ainda é incipiente a incorporação de práticas avaliativas no cotidiano da ESF, bem como o conhecimento sobre a qualidade desses serviços, particularmente no estado do Ceará, considerado vanguardista nas ações de APS no país.

Dessa forma, o objetivo deste estudo foi analisar a qualidade da infraestrutura e processo de trabalho da ESF nos municípios do Ceará entre 2012 e 2014, averiguando a existência de variáveis mediadoras da qualidade.

## MÉTODOS

Estudo transversal utilizando dados secundários da avaliação externa do 1º (2012) e 2º (2014) ciclo do PMAQ-AB referentes ao estado do Ceará, Brasil. Para essa avaliação são utilizados três instrumentos com foco na equipe da ESF: módulo I – referente à infraestrutura, com variáveis observadas diretamente na unidade de saúde; módulo II – relacionado ao processo de trabalho, em que as perguntas são respondidas por um membro da equipe da ESF (médico, enfermeiro ou dentista); módulo III – com questões relacionadas à satisfação do usuário, abrangendo sua percepção e satisfação quanto ao acesso e à utilização da ESF. No presente estudo, apenas os módulos I e II (infraestrutura e processo de trabalho) foram utilizados. Os questionários continham cerca de 450 perguntas relacionadas à infraestrutura e 750 ao processo de trabalho.

A coleta de dados foi coordenada por um grupo de pesquisadores de universidades e instituições de pesquisa responsáveis pela avaliação externa do PMAQ-AB, que treinou e acompanhou os entrevistadores de campo e supervisores de coleta de dados, incluindo coordenadores estaduais de pesquisa.

No primeiro ciclo de avaliação, participaram um total de 184 (100%) municípios, incluindo 911 equipes (46,5% das EqSF implantadas até 2012). Já no segundo ciclo, participaram 183 (99,4%) municípios, incluindo 1.711 equipes (74,3% da EqSF estabelecidas até 2014). Verificaram-se, no ano de 2012, mais observações de estrutura do que de processo de trabalho, pois nesse ano o Ministério da Saúde estava particularmente interessado em infraestrutura; portanto, o módulo referente a esse aspecto foi aplicado em todas as unidades básicas de saúde, mesmo naquelas que não aderiram ao PMAQ-AB. Dos 184 municípios, foram utilizados dados dos 183 (99,4%) cujas equipes foram avaliadas nos dois ciclos. Analisaram-se dados de 1.441 equipes para infraestrutura e de 800 equipes para processo de trabalho. Como algumas EqSF não responderam todas as questões dos módulos I e II, o número de equipes varia de acordo com o desfecho e ano de avaliação.

### Criação de Índices

Os índices do presente estudo foram criados com base no banco de dados nacional da avaliação externa do PMAQ-AB, originalmente utilizados para avaliar a ESF no Brasil. Para tanto, identificaram-se as variáveis similares incluídas nos ciclos 2012 e 2014. Os itens foram organizados em 20 grupos (10 para infraestrutura e 10 para processo de trabalho), com base nas diretrizes da ESF^12^ e temas de avaliação do PMAQ-AB^10^, excluindo-se aqueles com mais de 5.000 observações ausentes por ano de pesquisa. A aplicação de uma série de testes validou estes agrupamentos estruturados: função de correlação de pares, coeficiente alfa de Cronbach e análise fatorial. Adicionalmente, cada item foi escalado de 0 a 1 (1 = resultado mais positivo) e os itens dentro de um grupo foram calculados para formar o índice composto (IC). Além disso, a média de todos os IC na categoria geral criou três variáveis gerais de índice composto, duas para infraestrutura e uma para processo de trabalho. A Tabela 1 descreve os IC criados.

**Tabela 1:** Lista de variáveis que constituíram cada índice composto criado com base nas questões da avaliação externa do Programa Nacional de Melhoria do Acesso e da Qualidade da Atenção Básica em 2012 e 2014.

Nome do índice composto	Nº de questões	Descrição das questões
Medicamentos	47	Lista completa de 47 medicamentos.
Testes diagnósticos	4	Teste para *Plasmodium* (teste de esfregaço espesso); teste rápido de HIV; teste rápido de gravidez; teste rápido para sífilis.
Vacinas	12	Vacina oral contra rotavírus; tetravalente (2012) e pentavalente (2014); DPT (difteria, tétano e coqueluche); tríplice viral; pneumocócica 10-valente; pneumocócica (Salk e Sabin); pneumocócica 23-valente; meningocócica C; hepatite B; influenza sazonal; dupla adulto tipo dT; BCG ID; tríplice bacteriana.
Insumos/materiais	17	Cartão de vacinação; caderneta da gestante; caderneta da crianças; abaixador de língua em quantidade suficiente; agulhas descartáveis de vários tamanhos; ataduras; caixas térmicas para vacinas; fita métrica; espéculo descartável; equipo de soro macrogotas e microgotas; escovinha endocervical; espátula de Ayres; esparadrapo, fita micropore e outros; fixador de lâminas; gaze; lâmina de vidro com lado fosco; porta-lâminas ou frasco plástico com tampa para lâmina; tiras reagentes de medida de glicemia capilar; seringas descartáveis de diversos tamanhos; seringas descartáveis com agulha acoplada; recipiente duro para descarte de perfurocortantes.
Equipamentos médicos	21	Aparelho de pressão adulto; aparelho de pressão infantil; aparelho de nebulização; balança antropométrica de 200 kg; balança infantil; régua antropométrica; estetoscópio adulto; estetoscópio infantil; foco de luz para exame ginecológico; geladeira exclusiva para vacinas; geladeira exclusiva para farmácia; glicosímetro; mesa para exame ginecológico com perneira; mesa para exame clínico; oftalmoscópio; sonar; termômetro clínico; otoscópio; kit de monofilamento para teste de sensibilidade (estesiômetro); lanterna clínica; termômetro de cabo extensor.
Equipamentos de informática	10	Pelo menos um computador em condições de uso; pelo menos uma *webcam* em condições de uso; um conjunto de caixas de som de computador; um estabilizador em condições de uso; pelo menos um microfone em condições de uso; pelo menos uma impressora em condições de uso; pelo menos uma TV em condições de uso; acesso à internet; acesso da equipe ao Telessaúde; sala dedicada ao uso da internet.
Espaço adequado	17	Sanitário para usuários (masculino e feminino); banheiro para funcionários; sala de espera; sala de vacinas; consultório médico; consultório de dentista; sala de inalação; sala de procedimentos; sala de curativos; sala de observação; sala de esterilização; sala de atividades coletivas; boa ventilação e climatização; iluminação adequada; pisos e paredes de superfícies laváveis; boa acústica na unidade de saúde; consultórios com privacidade para os usuários.
Serviços ofertados	9	Veículo (visitas domiciliares e outras atividades externas); atendimento das necessidades da equipe pelo veículo; consulta médica; consulta de enfermagem; consulta odontológica; dispensação de medicamentos na farmácia; vacinação; acolhimento e outros.
Acessibilidade	4	Corredores adaptados para cadeira de rodas; todas as entradas externas e portas adaptadas para cadeira de rodas; cadeiras de rodas disponíveis para os usuários; banheiros para pessoas com deficiência.
Identificação na UBS	14	Sinalização adequada; horário de funcionamento da unidade de saúde de acordo com recomendações do Ministério da Saúde; listagem de atividades oferecidas pela equipe disponível aos usuários; escala dos profissionais com nome e horário de trabalho disponível aos usuários; divulgação aos usuários sobre participação da UBS no “Saúde Mais Perto de Você – Acesso e Qualidade (PMAQ)”; divulgação do telefone da ouvidoria do Ministério da Saúde; uso do crachá de identificação pelos profissionais; não divulgação das ações da equipe aos usuários; turnos de abertura da unidade (manhã, tarde e noite); abertura da unidade em todos os dias da semana (de segunda a sexta-feira); oferecimento de serviços nos finais de semana; funcionamento durante o horário de almoço.
Qualidade geral da infraestrutura I	10	Identificação na UBS; acessibilidade; serviços ofertados; espaço adequado; equipamentos de informática; equipamentos médicos; insumos/materiais; vacinas; testes diagnósticos; medicamentos.
Qualidade geral da infraestrutura II	9	Identificação na UBS; acessibilidade; serviços ofertados; espaço adequado; equipamentos de informática; insumos/materiais; vacinas; testes diagnósticos; medicamentos.
Vínculo profissional	6	Contrato com a administração pública direta; estabilidade no trabalho/obrigação de contratação; forma como conseguiu o emprego; plano de carreira e salários; recebimento de incentivo financeiro ou gratificação por desempenho; participação da equipe em processos de educação permanente organizados pelo município.
Planejamento	10	A equipe realiza planejamento das atividades mensalmente? A equipe realiza análise e monitoramento das informações e indicadores de saúde? A equipe realizou autoavaliação nos últimos seis meses? A equipe realiza reuniões ou encontros com frequência? Existe definição de área de cobertura da equipe? A equipe de saúde tem mapas do território? Os registros usados pela equipe são organizados por família? Existe um modelo-padrão para o preenchimento da folha de rosto dos prontuários médicos? Existe registro eletrônico implementado pela equipe de saúde? A equipe considera a visão do usuário para a reorganização e qualificação do processo de trabalho?
Apoio institucional	5	A equipe recebe apoio ou ajuda para o planejamento e organização do processo de trabalho? O município oferece à equipe de saúde informações que auxiliem na análise da situação de saúde da população? A equipe recebe apoio ou suporte para discussão dos dados e monitoramento do sistema de saúde? A equipe recebe apoio institucional permanente do município para discussão sobre processo de trabalho e ajuda nos problemas identificados? A equipe de saúde recebe ajuda de outros profissionais para auxiliar e/ou apoiar a resolução de casos complexos?
Acolhimento	7	A equipe realiza “acolhimento” à demanda espontânea na unidade de saúde? A equipe dispõe de serviço para remoção do usuário, quando necessária? A agenda da equipe de saúde é organizada para a realização de grupos de educação em saúde? A equipe realiza renovação de receitas para usuários de cuidado continuado ou de programas como os de hipertensão e diabetes, sem a necessidades de marcação de consultas médicas? Existe reserva de vagas na agenda ou horário de fácil acesso ao professional para que o usuário possa buscar e mostrar resultados de exames? Existe reserva de vagas na agenda ou horário de fácil acesso ao profissional para que o usuário possa sanar dúvidas pós-consulta ou mostrar como evoluiu sua situação? A equipe realiza encaminhamento de usuários com queixa de acuidade visual ou demanda de avaliação de refração, sem necessidade de marcação de consulta?
Exames	11	A equipe oferece ações para mulheres grávidas? A equipe oferece ações para crianças? A equipe oferece ações para pacientes com diabetes mellitus? A unidade de saúde realiza o exame de creatinina? A unidade de saúde realiza o exame de perfil lipídico? A unidade de saúde realiza o exame de eletrocardiograma? A unidade de saúde realiza o exame de hemoglobina glicosilada? A unidade de saúde realiza o exame de baciloscopia para tuberculose? A unidade de saúde realiza o exame de radiografia de tórax para diagnosticar tuberculose? A unidade de saúde realiza o exame de baciloscopia para hanseníase? A unidade de saúde realiza o exame de mamografia?
Pré-natal	14	A unidade de saúde realiza o exame de glicemia de jejum na rede de atenção pré-natal? A unidade de saúde realiza o exame de sorologia para sífilis (VDRL) na rede de atenção pré-natal? A unidade de saúde realiza o exame de sorologia para HIV na rede de atenção pré-natal? A unidade de saúde realiza o exame de sorologia para hepatite B na rede de atenção pré-natal? A unidade de saúde realiza o exame sumário e cultura de urina na rede de atenção pré-natal? A equipe alimenta mensalmente o sistema de informação do pré-natal? A equipe utiliza a caderneta ou cartão para acompanhamento das gestantes? Há registro sobre o profissional responsável pelo acompanhamento da gestante? Há registro sobre consulta odontológica da gestante? Há registro sobre o estado de vacinação da gestante? Há registro sobre coleta de exame citopatológico da gestante? A equipe orienta a gestante sobre vacinas contra o tétano? A equipe recebe os exames das gestantes do território em tempo oportuno para efetuar intervenções necessárias? É realizada aplicação de penicilina G benzatina na unidade de saúde?
Saúde da criança	9	A equipe realiza consulta de puericultura nas crianças de até dois anos? A equipe utiliza a caderneta de saúde da criança para o acompanhamento do crescimento e desenvolvimento? Tem espelho das cadernetas de saúde da criança ou outra ficha com informações equivalentes na unidade? No acompanhamento das crianças do território, há registro sobre vacinação em dia? No acompanhamento das crianças do território, há registro sobre crescimento e desenvolvimento? No acompanhamento das crianças do território, há registro sobre estado nutricional? No acompanhamento das crianças do território, há registro sobre teste do pezinho? No acompanhamento das crianças do território, há registro sobre violência familiar? No acompanhamento das crianças do território, há registro sobre acidentes?
Promoção da saúde	12	A equipe oferta ações educativas e de promoção da saúde direcionadas para mulheres (câncer do colo do útero e de mama)? A equipe oferta ações educativas e de promoção da saúde direcionadas para o planejamento familiar? A equipe oferta ações educativas e de promoção da saúde direcionadas para gestantes e puérperas (aleitamento materno)? A equipe oferta ações educativas e de promoção da saúde direcionadas para homens? A equipe oferta ações educativas e de promoção da saúde direcionadas para idosos? A equipe oferta ações educativas e de promoção da saúde direcionadas para a alimentação saudável? A equipe oferta ações educativas e de promoção da saúde direcionadas para estratégias educativas relacionadas à saúde sexual e à saúde reprodutiva? Realiza grupos com enfoque na orientação sobre doenças transmissíveis (como dengue, tuberculose, hanseníase, HIV e tracoma), conforme a necessidade do território? Realiza grupos com enfoque na orientação sobre o uso, abuso e dependência decorrentes do uso de crack, álcool e outras drogas? Realiza grupos com enfoque na orientação sobre o uso, abuso e dependência de ansiolíticos e benzodiazepínicos? A equipe aborda questões referentes ao sofrimento psíquico ou de promoção de saúde mental no território? A equipe incentiva e desenvolve práticas corporais e/ou atividades físicas na unidade básica de saúde e/ou no território?
Visita domiciliar	9	A equipe possui protocolo ou critérios para visita domiciliar? As famílias da área de abrangência da equipe de atenção básica são visitadas com periodicidade distinta, de acordo com avaliações de risco e vulnerabilidade? Os agentes comunitários de saúde têm a programação das visitas feita em função das prioridades de toda a equipe? A equipe possui levantamento/mapeamento dos usuários adscritos que necessitam receber cuidados no domicílio (exceto acamados)? A equipe possui registro do número de acamados e domiciliados do território? No cuidado domiciliar, os profissionais da equipe realizam atendimento clínico (usuário idoso e/ou que necessite de cuidado no domicílio)? No cuidado domiciliar, os profissionais da equipe realizam procedimentos de enfermagem? A equipe dispõe de canais de comunicação que permitem aos usuários expressarem suas demandas, reclamações e/ou sugestões na atenção básica? Há conselho local de saúde ou outros espaços de participação popular?
Saúde na escola	15	A equipe realiza atividades na escola? A equipe realiza atualização do calendário vacinal? A equipe realiza detecção precoce de hipertensão arterial sistêmica? A equipe realiza detecção de agravos de saúde negligenciados? A equipe realiza avaliação antropométrica? A equipe realiza avaliação oftalmológica? A equipe realiza avaliação nutricional? A equipe realiza avaliação da saúde bucal? A equipe realiza ações de segurança alimentar e promoção da alimentação saudável (atividades educativas sobre promoção da alimentação e modos de vida saudáveis)? A equipe realiza promoção das práticas corporais e atividade física nas escolas? A equipe realiza educação para a saúde sexual, saúde reprodutiva e prevenção das infecções sexualmente transmissíveis e aids? A equipe realiza ações de prevenção do uso de álcool, tabaco e outras drogas? A equipe realiza ações de capacitação dos profissionais de educação para trabalhar com educação para a saúde? A equipe realiza debates com os professores da escola? A equipe não realiza ações de promoção da saúde e prevenção?
Qualidade geral do processo de trabalho	10	Vínculo profissional; planejamento; apoio institucional; acolhimento; exames; pré-natal; saúde da criança; promoção da saúde; saúde na escola; visita domiciliar.

UBS: unidade básica de saúde

### Análise Estatística

O nível de avaliação é a ESF, mas a unidade de análise é o município. Assim, o IC do nível municipal foi criado pela média de todos os escores das EqSF do município. Dependendo do tamanho do município, o número de EqSF variou de muito poucas (menos de 5) a 120 equipes. Foram realizadas comparações para verificar se todos os IC, nos dois momentos, foram estatisticamente significantes, usando o teste t, Anova e teste T^2^ de Hotelling. O Anova e as equações de estimativa generalizada (GEE) foram usadas para comparar os IC nas diferentes regiões e tamanhos dos municípios.

Desse modo, a variação para cada IC foi criada com base no município, sendo calculada como o resultado do valor do IC de 2014 menos o valor do IC de 2012. Esses valores foram comparados com base na região e no porte populacional do município. Calculou-se, ainda, o percentual de mudança entre o 1º e o 2º ciclo para cada um dos IC.

### Variáveis de Estratificação

Repetiu-se a análise estatística usando duas variáveis de estratificação: tamanho do município (população) e região de saúde. Os municípios foram categorizados com base no número de habitantes, considerando-se as diretrizes do Instituto Brasileiro de Geografia e Estatística (IBGE): nível 1 (0 a 5.000), nível 2 (5.001 a 10.000), nível 3 (10.001 a 20.000), nível 4 (20.001 a 50.000), nível 5 (50.001 a 100.000), nível 6 (100.001 a 500.000) e nível 7 (acima de 500.001)^13^. Contudo, para a presente pesquisa, os níveis 1 e 2 foram agrupados. Essa estratificação foi adotada para testar a hipótese de que existem diferenças na melhoria de qualidade por porte populacional, haja vista que os desafios e a capacidade de gerenciamento diferem conforme o tamanho do município.

O estado do Ceará é dividido em cinco macrorregiões de saúde: Fortaleza (44 municípios), Sobral (54 municípios), Cariri (45 municípios), Sertão Central (20 municípios) e Litoral Leste/Jaguaribe (21 municípios). Considerou-se como hipótese que as melhorias de qualidade podem diferir nas regiões do estado, devido às características intrínsecas de cada uma. No processo de regionalização da saúde, foram criadas primeiro as macrorregiões de Fortaleza, Sobral e Cariri, consideradas os polos mais desenvolvidos, contando com rede de atenção especializada e hospitais terciários de referência em suas respectivas sedes. As macrorregiões Sertão Central e Litoral Leste/Jaguaribe foram criadas em 2011 e 2014, respectivamente, por desmembramento da macrorregião Fortaleza^14^.

## RESULTADOS

Constatou-se que a maioria (18 de 20) dos IC foi significativamente melhor no segundo ciclo do que no primeiro (Tabela 2). Apenas dois IC não mudaram significativamente ao longo do tempo, um relacionado à infraestrutura (vacinas disponíveis na unidade básica de saúde – UBS), e o outro relacionado ao processo de trabalho (exames).

**Tabela 2:** Comparação da variável índice composto nos anos avaliados. Ceará, 2012 e 2014.

Variáveis	n^a^	Ano	Média^b^	DP	p^c^	% de variação
Variáveis de infraestrutura						
Identificação na UBS	1.408	2012	0,54	0,16	< 0,001	16,67
	1.441	2014	0,63	0,13		
Acessibilidade	1.440	2012	0,22	0,29	0,001	136,36
	1.441	2014	0,52	0,34		
Serviços ofertados	1,441	2012	0,79	0,14	0,000	13,92
	1.441	2014	0,90	0,11		
Espaço adequado	1.428	2012	0,61	0,18	0,000	16,39
	1.441	2014	0,71	0,18		
Equipamentos de informática	1.438	2012	0,16	0,20	0,000	56,25
	1.441	2014	0,25			
Equipamentos médicos	448	2012	0,63	0,12	0,000	15,87
	1.441	2014	0,73	0,11		
Insumos/materiais	1.441	2012	0,88	0,11	0,000	6,81
	1.441	2014	0,94	0,08		
Vacinas	1.441	2012	0,82	0,15	0,421	1,21
	1.441	2014	0,83	0,13		
Testes diagnósticos	1.441	2012	0,04	0,12	0,000	413,5
	1.441	2014	0,19	0,34		
Medicamentos	1.441	2012	0,43	0,21	0,000	16,27
	1.441	2014	0,50	0,17		
Qualidade geral da infraestrutura I	433	2012	0,51	0,89	0,000	21,56
	1.441	2014	0,62	0,10		
Qualidade geral da infraestrutura II	1.392	2012	0,50	0,09	0,000	22,00
	1.441	2014	0,61	0,11		

Variáveis de processo de trabalho						
Vínculo profissional	782	2012	0,50	0,21	0,000	10,00
	789	2014	0,55	0,21		
Planejamento	784	2012	0,81	0,11	0,000	6,17
	786	2014	0,86	0,75		
Apoio institucional	698	2012	0,93	0,15	< 0,018	8,13
	798	2014	0,95	0,15		
Acolhimento	792	2012	0,80	0,18	0,000	8,75
	800	2014	0,87	0,15		
Exames	747	2012	0,96	0,74	0,521	0,00
	800	2014	0,96	0,90		
Pré-natal	792	2012	0,87	0,84	0,000	3,44
	800	2014	0,90	0,85		
Saúde da criança	799	2012	0,76	0,15	0,000	11,84
	800	2014	0,85	0,14		
Promoção da saúde	779	2012	0,56	0,20	0,000	25,00
	783	2014	0,70	0,22		
Saúde na escola	686	2012	0,56	0,24	0,000	42,85
	766	2014	0,80	0,18		
Visita domiciliar	797	2012	0,69	0,16	0,000	27,53
	800	2014	0,88	0,13		
Qualidade geral do processo de trabalho	553	2012	0,76	0,80	0,000	10,52
	734	2014	0,84	0,76		

DP: desvio-padrão; UBS: unidade básica de saúde

^a^ Equipes de saúde da família avaliadas em cada índice composto. Número de respostas das equipes: 15.670 para variáveis de processo de trabalho e 23.022 para infraestrutura.

^b^ Valor médio para cada índice composto (IC), sendo 1,00 o valor máximo.

^c^ Teste t avaliando se há diferença entre os valores de IC entre os dois anos (2012 e 2014).

O percentual de melhoria não foi homogêneo em todos os IC investigados, variando de 0,0 a 413,5% (Tabela 2). Observou-se relação negativa entre o percentual de mudança (entre os dois ciclos do PMAQ-AB) e o valor inicial (referente a 2012) da variável, na qual quanto menor o valor inicial do IC, maior a variação na qualidade entre 2012 e 2014. Isso foi observado quando todas as variáveis foram analisadas conjuntamente (r = -0,4843; p = 0,0192). Quando as variáveis de infraestrutura e processo de trabalho foram avaliadas separadamente, apenas o conjunto das variáveis de processo de trabalho demonstraram essa relação negativa estatisticamente significante (infraestrutura: r = -0,4624 e p = 0,1785; processo de trabalho: r = -0,7031 e p = 0,0233).

Ao estudar os valores dos IC para diferentes regiões e porte populacional dos municípios, observou-se que, de maneira geral, as variáveis apresentaram melhoras na qualidade média dos municípios no período, impactando na qualidade dos resultados da APS (Tabela 3 e Tabela 4).

**Tabela 3:** Comparação dos valores das variáveis de infraestrutura e processo de trabalho, segundo o porte populacional dos municípios com avaliação externa do Programa Nacional de Melhoria do Acesso e da Qualidade da Atenção Básica. Ceará, 2012 e 2014. (Continua)

Variáveis de infraestrutura por porte populacional do município						Variáveis de processo de trabalho por porte populacional do município					
	
Variável	Porte populacional (em habitantes)	2012	2014	% mudança	p^a^	Variável	Porte populacional (em habitantes)	2012	2014	% mudança	p^a^
Identificação na UBS	0–10.000	0,542	0,633	16,78		Visita domiciliar	0–10.000	0,663	0,848	27,90	
	10.001–20.000	0,524	0,624	19,08			10.001–20.000	0,682	0,875	28,29	
	20.001–50.000	0,532	0,641	20,48			20.001–50.000	0,680	0,868	27,64	
	50.001–100.000	0,528	0,609	15,34	0,688		50.001–100.000	0,697	0,884	26,82	0,235
	100.001–500.000	0,587	0,686	16,86			100.001–500.000	0,741	0,931	25,64	
	500.001 ou +	0,534	0,600	12,35			500.001 ou +	0,707	0,740	4,67	

Acessibilidade	0–10.000	0,270	0,632	134,07		Saúde na escola	0–10.000	0,633	0,795	25,59	
	10.001–20.000	0,200	0,517	158,50			10.001–20.000	0,605	0,808	33,55	
	20.001–50.000	0,194	0,499	157,21			20.001–50.000	0,558	0,796	42,65	
	50.001–100.000	0,218	0,496	127,52	0,256		50.001–100.000	0,440	0,771	75,23	0,137
	100.001–500.000	0,279	0,559	100,35			100.001–500.000	0,553	0,858	55,15	
	500.001 ou +	0,233	0,600	157,51			500.001 ou +	0,577	0,555	-3,81	

Serviços ofertados	0–10.000	0,831	0,926	11,43		Promoção da saúde	0–10.000	0,615	0,638	3,74	
	10.001–20.000	0,770	0,900	16,88			10.001–20.000	0,571	0,684	19,79	
	20.001–50.000	0,797	0,898	12,67			20.001–50.000	0,556	0,708	27,34	
	50.001–100.000	0,769	0,899	16,90	0,562		50.001–100.000	0,504	0,676	34,13	0,971
	100.001–500.000	0,815	0,918	12,63			100.001–500.000	0,641	0,781	21,84	
	500.001 ou +	0,866	0,895	3,34			500.001 ou +	0,569	0,354	-37,78	

Espaço adequado	0–10.000	0,616	0,717	16,39		Vínculo profissional	0–10.000	0,419	0,462	10,26	
	10.001–20.000	0,579	0,683	17,96			10.001–20.000	0,423	0,493	16,55	
	20.001–50.000	0,584	0,725	24,14			20.001–50.000	0,548	0,600	9,49	
	50.001–100.000	0,625	0,681	8,96	0,285		50.001–100.000	0,498	0,536	7,63	0,001
	100.001–500.000	0,653	0,762	16,69			100.001–500.000	0,473	0,552	16,70	
	500.001 ou +	0,718	0,658	-8,35			500.001 ou +	0,823	0,770	-6,43	

Equipamentos de informática	0–10.000	0,220	0,328	49,09		Planejamento	0–10.000	0,809	0,857	5,93	
	10.001–20.000	0,103	0,227	120,38			10.001–20.000	0,809	0,852	5,31	
	20.001–50.000	0,126	0,243	92,85			20.001–50.000	0,818	0,868	6,11	
	50.001–100.000	0,140	0,220	57,14	0,920		50.001–100.000	0,775	0,851	9,80	0,688
	100.001–500.000	0,311	0,347	11,57			100.001–500.000	0,855	0,891	4,21	
	500.001 ou +	0,396	0,390	-1,51			500.001 ou +	0,858	0,663	-22,72	

Equipamentos médicos	0–10.000	0,650	0,767	18,00		Apoio institucional	0–10.000	0,930	0,963	3,55	
	10.001–20.000	0,611	0,720	17,83			10.001–20.000	0,923	0,946	2,49	
	20.001–50.000	0,643	0,732	13,84			20.001–50.000	0,948	0,960	1,26	
	50.001–100.000	0,622	0,708	13,82	0,980		50.001–100.000	0,910	0,952	4,61	0,407
	100.001–500.000	0,692	0,780	12,71			100.001–500.000	0,937	0,986	5,23	
	500.001 ou +	0,780	0,717	-8,07			500.001 ou +	0,758	0,418	-44,85	

Insumos/materiais	0–10.000	0,889	0,945	6,29		Acolhimento	0–10.000	0,818	0,878	7,33	
	10.001–20.000	0,872	0,939	7,68			10.001–20.000	0,809	0,860	6,30	
	20.001–50.000	0,880	0,944	7,27			20.001–50.000	0,805	0,866	7,58	
	50.001–100.000	0,877	0,938	6,95	0,725		50.001–100.000	0,757	0,857	13,21	0,212
	100.001–500.000	0,911	0,954	4,72			100.001–500.000	0,811	0,905	11,59	
	500.001 ou +	0,875	0,932	6,51			500.001 ou +	0,845	0,678	-19,76	

Vacinas	0–10.000	0,829	0,833	0,48		Exames	0–10.000	0,931	0,947	1,72	
	10.001–20.000	0,825	0,831	0,72			10.001–20.000	0,951	0,942	-0,95	
	20.001–50.000	0,796	0,821	3,14			20.001–50.000	0,958	0,957	-0,10	
	50.001–100.000	0,826	0,814	-1,45	0,996		50.001–100.000	0,954	0,955	0,10	0,037
	100.001–500.000	0,853	0,852	-0,11			100.001–500.000	0,986	0,986	0	
	500.001 ou +	0,891	0,801	-10,10			500001 ou +	0,992	0,893	-9,98	

Testes diagnósticos	0–10.000	0,034	0,091	167,64		Pré-natal	0–10.000	0,872	0,905	3,78	
	10.001–20.000	0,012	0,158	1216,67			10.001–20.000	0,872	0,904	3,67	
	20.001–50.000	0,030	0,067	123,33			20.001–50.000	0,857	0,901	5,13	
	50.001–100.000	0,015	0,246	1540,00	0,036		50.001–100.000	0,861	0,888	3,13	0,693
	100.001–500.000	0,112	0,460	310,71			100.001–500.000	0,901	0,928	2,99	
	500.001 ou +	0,105	0,305	190,47			500.001 ou +	0,892	0,815	-8,63	

Medicamentos	0–10.000	0,443	0,462	4,28		Saúde da criança	0–10.000	0,730	0,774	6,03	
	10.001–20.000	0,439	0,465	9,92			10.001–20.000	0,744	0,851	14,38	
	20.001–50.000	0,449	0,502	11,80			20.001–50.000	0,751	0,843	12,25	
	50.001–100.000	0,425	0,480	12,94	0,696		50.001–100.000	0,718	0,807	12,39	0,036
	100.001–500.000	0,393	0,546	38,93			100.001–500.000	0,834	0,935	12,11	
	500.001 ou +	0,154	0,598	288,31			500.001 ou +	0,842	0,777	-7,71	

Qualidade geral da infraestrutura I	0–10.000	0,530	0,633	19,43		Qualidade geral do processo de trabalho	0–10.000	0,754	0,822	9,02	
	10.001–20.000	0,489	0,606	23,92			10.001–20.000	0,752	0,824	9,57	
	20.001–50.000	0,507	0,607	19,72	0,374		20.001–50.000	0,763	0,839	9,96	
	50.001–100.000	0,513	0,609	18,71			50.001–100.000	0,725	0,822	13,38	0,338
	100.001–500.000	0,579	0,686	18,48			100.001–500.000	0,794	0,876	10,33	
	500.001 ou +	0,603	0,650	7,79			500.001 ou +	0,795	0,702	-11,69	

Qualidade geral da infraestrutura II	0–10.000	0,524	0,619	18,12							
	10.001–20.000	0,480	0,594	23,75							
	20.001–50.000	0,487	0,593	21,76	0,846						
	50.001–100.000	0,491	0,598	21,79							
	100.001–500.000	0,541	0,676	24,95							
	500.001 ou +	0,527	0,642	21,82							

UBS: unidade básica de saúde

^a^ Anova - Equações de estimativa generalizada.

**Tabela 4:** Comparação dos valores das variáveis de infraestrutura e processo de trabalho, segundo a região de saúde na avaliação externa do Programa Nacional de Melhoria do Acesso e da Qualidade da Atenção Básica. Ceará, 2012 e 2014. (Continua)

Variáveis de infraestrutura por região						Variáveis de processo de trabalho por região										

Variável	Região de saúde	2012	2014	% mudança	p^a^	Variável	Região de saúde		2012		2014		% mudança		p^a^	
Identificação na UBS	Fortaleza	0,510	0,617	20,98		Vínculo profissional	Fortaleza		0,485		0,558		15,05			
	Sobral	0,494	0,596	20,64			Sobral		0,526		0,531		0,95			
	Cariri	0,618	0,685	10,84	0,002		Cariri		0,514		0,590		14,78		0,673	
	Sertão Central	0,476	0,609	27,94			Sertão Central		0,371		0,479		29,11			
	Litoral Leste/Jaguaribe	0,583	0,677	16,12			Litoral Leste/Jaguaribe		0,514		0,532		3,50			

Acessibilidade	Fortaleza	0,253	0,525	107,50		Planejamento	Fortaleza		0,786		0,844		7,37			
	Sobral	0,184	0,475	158,15			Sobral		0,809		0,858		6,05			
	Cariri	0,214	0,571	166,82	0,894		Cariri		0,837		0,878		4,89		0,016	
	Sertão Central	0,155	0,501	223,22			Sertão Central		0,787		0,878		11,56			
	Litoral Leste/Jaguaribe	0,261	0,461	76,62			Litoral Leste/Jaguaribe		0,846		0,847		0,11			

Serviços ofertados	Fortaleza	0,786	0,903	14,88		Apoio institucional	Fortaleza		0,889		0,915		2,92			
	Sobral	0,780	0,890	14,10			Sobral		0,952		0,969		1,78			
	Cariri	0,812	0,916	12,80	0,503		Cariri		0,937		0,969		3,41		0,043	
	Sertão Central	0,745	0,917	23,08			Sertão Central		0,936		0,953		1,81			
	Litoral Leste/Jaguaribe	0,788	0,880	11,67			Litoral Leste/Jaguaribe		0,948		0,968		2,10			

Espaço adequado	Fortaleza	0,639	0,701	9,70		Acolhimento	Fortaleza		0,803		0,844		5,10			
	Sobral	0,594	0,688	15,82			Sobral		0,771		0,837		8,56			
	Cariri	0,595	0,758	27,39	0,343		Cariri		0,813		0,879		8,11		0,001	
	Sertão Central	0,582	0,708	21,64			Sertão Central		0,778		0,906		16,45			
	Litoral Leste/Jaguaribe	0,597	0,649	8,71			Litoral Leste/Jaguaribe		0,809		0,936		15,69			

Equipamentos de informática	Fortaleza	0,191	0,243	27,22		Exames	Fortaleza		0,969		0,944		-2,57			
	Sobral	0,151	0,263	74,17			Sobral		0,956		0,952		-0,41			
	Cariri	0,134	0,265	97,76	0,266		Cariri		0,967		0,980		1,34		0,310	
	Sertão Central	0,141	0,250	77,30			Sertão Central		0,930		0,961		3,33			
	Litoral Leste/Jaguaribe	0,186	0,256	37,63			Litoral Leste/Jaguaribe		0,946		0,935		-1,16			

Equipamentos médicos	Fortaleza	0,653	0,720	10,26		Pré-natal	Fortaleza		0,879		0,902		2,61			
	Sobral	0,626	0,710	13,41			Sobral		0,879		0,902		2,61			
	Cariri	0,644	0,754	17,08	0,017		Cariri		0,853		0,891		4,45		0,568	
	Sertão Central	0,567	0,719	26,80			Sertão Central		0,867		0,909		4,84			
	Litoral Leste/Jaguaribe	0,657	0,773	17,65			Litoral Leste/Jaguaribe		0,870		0,924		6,20			

Insumos/materiais	Fortaleza	0,894	0,936	4,69		Saúde da criança	Fortaleza		0,776		0,861		10,95			
	Sobral	0,883	0,941	6,56			Sobral		0,756		0,833		10,18			
	Cariri	0,887	0,958	8,00	0,363		Cariri		0,767		0,862		12,38		0,058	
	Sertão Central	0,840	0,930	10,71			Sertão Central		0,704		0,850		20,73			
	Litoral Leste/Jaguaribe	0,877	0,938	6,95			Litoral Leste/Jaguaribe		0,706		0,792		12,18			

Vacinas	Fortaleza	0,840	0,838	-0,23		Promoção da saúde	Fortaleza		0,514		0,664		29.18			
	Sobral	0,788	0,797	1,14			Sobral		0,610		0,693		13,60			
	Cariri	0,839	0,830	-1,07	0,666		Cariri		0,608		0,718		18,09		0,023	
	Sertão Central	0,798	0,840	5,26			Sertão Central		0,463		0,733		58,31			
	Litoral Leste/Jaguaribe	0,818	0,827	1,10			Litoral Leste/Jaguaribe		0,556		0,751		35,07			

Testes diagnósticos	Fortaleza	0,039	0,200	412,82		Saúde na escola	Fortaleza		0,524		0,772		47,32			
	Sobral	0,047	0,234	397,87			Sobral		0,561		0,766		36,54			
	Cariri	0,025	0,085	240,00	0,497		Cariri		0,615		0,826		34,30		0,001	
	Sertão Central	0,016	0,246	1.437,50			Sertão Central		0,493		0,829		68,15			
	Litoral Leste/Jaguaribe	0,058	0,352	506,89			Litoral Leste/Jaguaribe		0,579		0,849		46,63			

Medicamentos	Fortaleza	0,449	0,495	10,24		Visita domiciliar	Fortaleza		0,706		0,882		24,92			
	Sobral	0,353	0,406	15,01			Sobral		0,669		0,834		24,66			
	Cariri	0,428	0,517	20,79	0,045		Cariri		0,692		0,896		29,47		0,042	
	Sertão Central	0,447	0,498	11,40			Sertão Central		0,695		0,910		30,93			
	Litoral Leste/Jaguaribe	0,499	0,498	-0,20			Litoral Leste/Jaguaribe		0,727		0,921		26,68			

Qualidade geral da infraestrutura I	Fortaleza	0,534	0,618	15,73		Qualidade geral do processo de trabalho	Fortaleza		0,750		0,822		9,60			
	Sobral	0,482	0,607	25,93			Sobral		0,772		0,823		6,60			
	Cariri	0,523	0,634	21,22	0,160		Cariri		0,771		0,851		10,37		0,171	
	Sertão Central	0,481	0,622	29,31			Sertão Central		0,711		0,848		19,26			
	Litoral Leste/Jaguaribe	0,531	0,631	18,83			Litoral Leste/Jaguaribe		0,757		0,844		11,49			

Qualidade geral da infraestrutura II	Fortaleza	0,509	0,606	19,05												
	Sobral	0,475	0,595	25,26												
	Cariri	0,505	0,620	22,77	0,154											
	Sertão Central	0,467	0,611	30,83												
	Litoral Leste/Jaguaribe	0,519	0,615	18,49												

UBS: unidade básica de saúde

^a^ Anova - Equações de estimativa generalizada.

Ao observar mudanças de qualidade na infraestrutura, com base no tamanho do município, somente o IC de testes diagnósticos apresentou percentuais de mudança significativamente diferentes entre os grupos (p = 0,036), ocorrendo maior impacto positivo na melhoria da qualidade nos municípios com população de 10.001 a 20.000 habitantes e de 50.001 a 100.000 habitantes. Esses dados demonstram que o tamanho do município não pareceu influenciar na melhoria da qualidade das variáveis de infraestrutura durante o período estudado.

Constatou-se que poucos IC de infraestrutura foram influenciados pela região de saúde do município, com apenas três apresentando mudanças estatisticamente significativas: identificação na UBS (p = 0,002), equipamentos médicos (p=0,017) e medicamentos (p = 0,045). Contudo, apesar de não haver diferenças estatisticamente significantes entre as regiões de saúde, observou-se que a região Sertão Central apresentou maior percentual de melhorias na maior parte das variáveis de infraestrutura analisadas, quais sejam: identificação na UBS, acessibilidade, serviços ofertados, equipamentos de informática, equipamentos médicos, insumos/materiais, vacinas, testes diagnósticos, medicamentos e infraestrutura geral I e II (Tabela 4).

Ao se observar as variáveis de IC relacionadas ao processo de trabalho, os diferentes portes populacionais de município foram associados significativamente à mudança na melhoria da qualidade em três IC avaliados, em relação ao vínculo profissional (p = 0,001), exames (p = 0,037) e saúde da criança (p = 0,036). Destaca-se que, apesar de nem sempre ser um achado estatisticamente significante, as variáveis promoção da saúde, saúde na escola, planejamento, acolhimento e qualidade geral do processo de trabalho apresentaram maior variação percentual nos municípios de 50.001 a 100.000 habitantes. Interessante observar que municípios com população acima de 500.000 habitantes apresentaram variação negativa ao longo dos anos em todos os IC de processo de trabalho, com exceção da visita domiciliar (Tabela 3).

Ao observar as mudanças no processo de trabalho por região (Tabela 4), ocorreu variação significativa entre elas em seis IC estudados: planejamento (p = 0,016), apoio institucional (p = 0,043), acolhimento (p = 0,001), promoção da saúde (p = 0,023), saúde na escola (p = 0,001) e visita domiciliar (p = 0,042). O maior aumento dos IC ocorreu na região Sertão Central, com maior variação positiva entre as regiões e em 9 dos 11 IC estudados, enquanto Litoral Leste/Jaguaribe e Cariri apresentaram maiores variações em um IC. Interessante observar que os valores de IC em 2012 para a região Sertão Central foram, em geral, os mais baixos entre as diferentes regiões.

## DISCUSSÃO

Este é o primeiro artigo que, por meio de índices compostos, avalia a qualidade da ESF no estado do Ceará, nas dimensões infraestrutura e processo de trabalho, utilizando dados da avaliação externa do PMAQ-AB do 1º e 2º ciclo (2012 e 2014). No geral, observou-se que ocorreu variação positiva dos IC de infraestrutura e processo de trabalho (mudança significativa em 18 dos 20 IC avaliados), o que indica melhoria na qualidade da ESF no período estudado. Investigou-se, também, onde essa melhoria ocorreu de forma mais intensa e verificou-se uma relação inversa entre resultado do IC em 2012 e o percentual de mudança (diferença de valores entre 2014 e 2012) – ou seja, quanto menor o valor em 2012, maior a melhoria da variável. Isso demonstra uma desejável melhora equitativa dos IC no período.

Esse desempenho parece refletir, em alguma medida, a indução realizada por políticas de avaliação e monitoramento, com ampliação de investimentos e uso adequado de recursos no atendimento de demandas da APS^8,15^, assim como o programa de requalificação da infraestrutura das unidades básicas de saúde do país (Requalifica UBS)^16^. Estudo semelhante realizado em todo o Brasil também demonstrou melhoria equitativa nos indicadores analisados, sobretudo quando avaliando as regiões Norte e Nordeste^17^. Interessante observar que, diferentemente do estudo de abrangência nacional, que apresentou relação inversa mais proeminente nos IC relacionados à infraestrutura^17^, neste trabalho foi observada tal relação mais forte nos IC relacionados aos processos de trabalho. Isso pode significar que, de uma maneira geral, o Requalifica UBS^16^ foi efetivo, mas que, no estado do Ceará, a atuação das equipes, bem como os processos de gestão a elas vinculados, foi capaz de responder mais fortemente que em outras regiões do país no tocante aos processos de trabalho.

Convém assinalar que essa relação inversa mais proeminente nos IC referentes ao processo de trabalho pode ser resultante do protagonismo das equipes, que parecem ter trabalhado essas questões de forma mais efetiva, minimizando as diferenças de qualidade entre as variáveis estudadas e qualificando o trabalho da ESF de forma mais equânime. De forma geral, as equipes têm mais autonomia para atuar nos problemas relacionados ao processo de trabalho do que na dimensão da infraestrutura, que demanda recursos financeiros nem sempre disponíveis^12,15^. Assim, observa-se que as equipes participantes do PMAQ-AB efetivamente ampliaram seu escopo de práticas, apoiadas pela gestão municipal. Dessa forma, avançaram nas mudanças relacionadas ao processo de trabalho para qualificar a ESF, tomando para si este processo de transformação, realizando autoavaliação e planejamento, traçando metas a serem implementadas conjuntamente pelas equipes.

Reconhece-se que a disponibilidade de recursos financeiros tem capacidade de induzir mais rapidamente melhorias na infraestrutura, enquanto as transformações de processo de trabalho demandam mais tempo, haja vista que requisitam mudanças na cultura organizacional, corresponsabilização de gestores e profissionais, além de reorganização das práticas de saúde^18^. O fato de os municípios cearenses terem conseguido efetivar avanços na organização de processos de trabalho de suas EqSF pode ser reflexo da atuação do estado. O protagonismo dele está associado aos processos de educação permanente e ao monitoramento dos indicadores desenvolvidos, sendo essas ações executadas como estratégia de consolidação do processo de regionalização^14^. Destarte, com o PMAQ, o Brasil assume a responsabilidade de gerenciar adequadamente a oferta de serviços, de forma que os resultados alcançados correspondam às metas estabelecidas ou às reais necessidades da população com uma política de incentivo programada^10^.

Os menores percentuais de mudança ocorreram nas variáveis exames, vacinas e pré-natal, que estão entre os IC com valores iniciais mais elevados. Adicionalmente, é importante compreender que a oferta de vacinas tem sua estrutura de logística organizada nacionalmente pelo Programa Nacional de Imunização (PNI)^16^. Assim, os imunobiológicos são adquiridos pelo governo federal, cabendo ao nível local/municipal a adequação das unidades conforme as normas técnicas do Ministério da Saúde e aplicação na população^19,20^. Dessa maneira, os municípios têm baixa interferência nessa variável, o que pode ser verificado pela não influência do porte populacional e região de saúde nesse IC.

É interessante observar que o maior aumento de IC ocorreu em testes diagnósticos, variável em que os municípios também têm pouca influência. A aquisição dos testes é realizada pelo Ministério da Saúde, mas compete aos estados e municípios sua efetiva estruturação e organização. A implantação dos testes rápidos e exames para diagnóstico de gravidez, de infecção pelo HIV e triagem de sífilis e hepatites virais na APS forma o conjunto de estratégias do Ministério da Saúde que tem como objetivo a qualificação e a ampliação do acesso da população brasileira à saúde^21^. Os dados demonstram que houve uma significativa melhora nesse indicador no estado, muito provavelmente devido ao aumento na aquisição dos insumos por parte do governo federal, mas também pela melhor organização do estado e dos municípios na distribuição e utilização desses insumos. Contudo, diferentemente da variável vacinas, o porte populacional e a região de saúde tiveram influência na melhora em testes diagnósticos. A explicação para isso não é muito clara, mas nos parece ter relação com a forma com que esses municípios se organizam para efetivar as ações relacionadas.

Observa-se, ainda, que a variação dos IC não ocorreu de forma homogênea nos grupos estudados, parecendo ser influenciada pelo porte populacional e pela regionalização. As maiores variações ocorreram nos municípios de menor porte populacional, localizados na região do Sertão Central e com menores valores de IC no primeiro ciclo de avaliação externa.

No processo de implantação da macrorregião Sertão Central, em 2011, sendo a penúltima instalada no estado do Ceará^14^, é possível que sua organização – mediante a construção do Plano Diretor Regional, viabilizada por meio de oficinas, reuniões, treinamentos e pactuações entres gestores municipais e destes com o gestor estadual – tenha mobilizado esforços de dirigentes e profissionais voltados à qualificação de seus processos de trabalho e infraestrutura, refletindo nos melhores IC da região no 2° ciclo II do PMAQ. Tal fato vem corroborar o efeito das questões regionais na implantação de políticas nacionais.

Ressalta-se que variáveis mediadoras devem ser consideradas na implantação de políticas públicas de saúde. Diferenças inter e intrarregionais nos sistemas de saúde podem ocorrer por diversas razões, sejam elas econômicas, culturais, educacionais, organizacionais, infraestruturais ou de perfil populacional, incluindo o epidemiológico e o demográfico^17^.

Em municípios com mais de 500.000 habitantes, caso da capital do estado, foi observada variação negativa nos resultados de IC referentes à qualidade do processo de trabalho. Esse fato pode ser efeito da mudança organizacional implementada na APS do município desde 2013, notadamente no processo de trabalho das EqSF. Nesse âmbito, destacam-se: mudanças de gerentes de unidades básicas de saúde; mudança na jornada de trabalho dos profissionais, que passaram a trabalhar em horários corridos de seis horas, gerando desencontros entre os membros das equipes; desestímulo ao planejamento local na ESF; mudanças na regulação do acesso dos usuários, com densa agenda de atendimento pela demanda espontânea; dentre outras^22,23^. Tais modificações parecem ter, até certo ponto, desarticulado as EqSF, distanciando-as do que é preconizado pela Política Nacional de Atenção Básica (PNAB)^12^. Estudos anteriores demonstraram relação entre índice de desenvolvimento humano (IDH), cobertura de ESF, cobertura do Programa Bolsa Família, tamanho populacional, indicadores de planejamento da ESF e de apoio institucional para ações da ESF e oferta de pré-natal e de exames pela ESF como variáveis que influenciam indicadores de saúde^24-26^. Portanto, apreende-se que, se o intuito é melhorar a qualidade da atenção à saúde, há necessidade de se empreender esforços voltados ao planejamento e ao apoio institucional, alinhados à missão organizacional, considerando os interesses do coletivo de trabalhadores, com vistas a assegurar a oferta de serviços e ações resolutivas.

Considerando-se o fato de que a maior variação positiva na melhoria da qualidade ocorreu em municípios e/ou regiões com menores IC iniciais, denota-se que a implementação do PMAQ-AB no Ceará induziu a qualificação da ESF de modo equitativo. Com efeito, essa característica também parece ocorrer no restante do país, e em outras políticas pautadas no princípio da equidade, como a PNAB, que também tem proporcionado redução de desigualdades, beneficiando municípios mais pobres, menores e com menor densidade demográfica^17,24,27^.

Reconhece-se que a implementação do PMAQ-AB exigiu maior protagonismo de gestores e trabalhadores na reestruturação das unidades básicas de saúde e nos processos de trabalho na ESF do que tradicionalmente ocorria nos estados brasileiros. Sabe-se que os padrões de acesso e qualidade são ressignificados em função da realidade concreta, do contexto, das prioridades, dos interesses e da negociação com os atores locais^8,28^. Nesse sentido, políticas de saúde indutoras da avaliação e monitoramento também parecem ter influência do contexto onde são implementadas^29^, devendo ser levadas em consideração na implantação de políticas públicas nacionais, mas com implementação local. Acredita-se que parte das diferenças observadas entre as regiões de saúde e porte populacional pode ter se dado em decorrência de questões regionais. Assim, para a plena implantação de políticas nacionais, é necessário apoio adicional às regiões que necessitam de maior incentivo para alcançar melhoria na qualidade. É importante frisar que, apesar da análise da presente pesquisa ter sido realizada no estado do Ceará, suas inferências, relacionadas à importância das questões de contexto na implantação de políticas, podem ser extrapoladas a outras partes do país.

O estudo reconhece que, mediante os IC avaliados, o PMAQ-AB, apesar de recente, fomenta a responsabilidade de gerenciar adequadamente a oferta de serviços de forma que as metas estabelecidas e as reais necessidades de saúde da população sejam atendidas e alcançadas com uma política de incentivo programada que impacte diretamente no financiamento, gestão da rede de serviços, apoio institucional, planejamento e organização de processos de trabalho.

## CONCLUSÃO

Melhorias de qualidade referentes à infraestrutura e ao processo de trabalho ocorreram de forma equitativa no período de implantação do PMAQ-AB no estado do Ceará. Apesar de a implementação do programa ter ocorrido quase universalmente entre os municípios do estado, os resultados dessa política não foram homogêneos, tendo sido influenciados pelo porte populacional e região de saúde. Observou-se que as políticas públicas são apropriadas e adaptadas de acordo com a realidade e/ou contexto em que são implementadas, com a flexibilidade de considerar a dinâmica e a complexidade dos territórios. Assim, esses aspectos devem ser levados em consideração quando políticas nacionais são implementadas localmente.
